# Enhancing the landscape of colorectal cancer using targeted deep sequencing

**DOI:** 10.1038/s41598-021-87486-3

**Published:** 2021-04-14

**Authors:** Chul Seung Lee, In Hye Song, Ahwon Lee, Jun Kang, Yoon Suk Lee, In Kyu Lee, Young Soo Song, Sung Hak Lee

**Affiliations:** 1 Department of Surgery, Seoul St. Mary’s Hospital, College of Medicine, The Catholic University of Korea, Seoul, Republic of Korea; 2 Department of Hospital Pathology, Seoul St. Mary’s Hospital, College of Medicine, The Catholic University of Korea, 222, Banpo-daero, Seocho-gu, Seoul, 06591 Republic of Korea; 3 Department of Pathology, College of Medicine, Konyang University, 158 Gwanjeodong-ro, Seo-gu, Daejeon, 35365 Republic of Korea

**Keywords:** Cancer, Genetics, Oncology

## Abstract

Targeted next-generation sequencing (NGS) technology detects specific mutations that can provide treatment opportunities for colorectal cancer (CRC) patients. We included 145 CRC patients who underwent surgery. We analyzed the mutation frequencies of common actionable genes and their association with clinicopathological characteristics and oncologic outcomes using targeted NGS. Approximately 97.9% (142) of patients showed somatic mutations. Frequent mutations were observed in TP53 (70%), APC (60%), and KRAS (49%). TP53 mutations were significantly linked to higher overall stage (p = 0.038) and lower disease-free survival (DFS) (p = 0.039). ATM mutation was significantly associated with higher tumor stage (p = 0.012) and shorter overall survival (OS) (p = 0.041). Stage 3 and 4 patients with ATM mutations (p = 0.023) had shorter OS, and FBXW7 mutation was significantly associated with shorter DFS (p = 0.002). However, the OS of patients with or without TP53, RAS, APC, PIK3CA, and SMAD4 mutations did not differ significantly (p = 0.59, 0.72, 0.059, 0.25, and 0.12, respectively). Similarly, the DFS between patients with RAS, APC, PIK3CA, and SMAD4 mutations and those with wild-type were not statistically different (p = 0.3, 0.79, 0.13, and 0.59, respectively). In multivariate Cox regression analysis, ATM mutation was an independent biomarker for poor prognosis of OS (p = 0.043). A comprehensive analysis of the molecular markers for CRC can provide insights into the mechanisms underlying disease progression and help optimize a personalized therapy.

## Introduction

Colorectal cancer (CRC) is the third most common malignancy worldwide^1^. Despite the advancements in CRC treatment and the decline in mortality rate over the past few decades, CRC remains the second most common cause of cancer death in women and third common cause of cancer death in men in Korea^2^. Although patients with localized stage CRC have a 5-year overall survival (OS) of 90%, cancer spread to distant organs carries a significantly worse prognosis with a 5-year OS of 14%^1^. Disease spread to distant organs is the major cause of morbidity and mortality in patients with CRC^3^.

Several genomic alterations, including KRAS, NRAS, and BRAF mutations, are associated with resistance to targeted therapy with epidermal growth factor receptor (EGFR) monoclonal antibodies, providing a molecular basis for selecting appropriate agents in the treatment of metastatic CRC^4^. Subsequent studies identified other genetic mutations of the EGFR signaling pathways involving the HER2 and FGFR1 genes^5^. Genomic analysis showed that alterations in p53, WNT–β-catenin, TGF-β, EGFR, and downstream MAPK/ERK and PI3K/Akt signaling pathways are associated with CRC tumorigenesis^6^. In the era of personalized medicine, an in-depth understanding of the molecular profiles and altered signaling pathways is important to identify the patients who may be able to benefit from such treatments.

Genetic or epigenetic alterations of the DNA mismatch repair (MMR) genes may have a predictive value in some cases with CRC. Although testing for MMR status in patients with CRC has been recommended as a workup test to evaluate the possible occurrence of Lynch syndrome, recent data revealed that microsatellite instability (MSI) is a predictive biomarker for immunotherapy^7^.

The next-generation sequencing (NGS) approach allows the agnostic analysis of large portions of the genome and can identify multiple mutations with increased sensitivity^8^. This method is currently used in pathology laboratories as a routine molecular test modality^9^. Combined with various clinical information and advanced bioinformatic analysis, the NGS data could be used as a basis for establishing a personalized treatment for cancer patients.

We aimed to describe the mutational profile of patients with CRCs using the targeted NGS approach and analyze their potential correlations with clinicopathological factors. In addition, we aimed to assess the biological and clinical significance of low variant allele frequency (VAF) for small variants and compare them with those of The Cancer Genome Atlas (TCGA) and Memorial Sloan Kettering Cancer Center (MSKCC) dataset, a publicly available archive.

## Results

### Study population

A total of 145 patients (76 men and 69 women) with CRC were included in this study. The median age was 60.9 years (range, 25–88 years). Ninety-two (63.4%) of the tumors occurred in the colon, while the remaining 53 (36.6%) tumors developed in the rectum. The detailed clinicopathological characteristics of the study cohort are shown in Table 1.

**Table 1:** Clinicopathological data of 145 colorectal cancer patients.

Clinicopathological parameters		Number of patients (N = 145, %)
Sex	Male	76 (52.4)
	Female	69 (47.6)
Age	≥ 60	78 (53.8)
	< 60	67 (46.2)
Tumor site	Right colon	45 (31.0)
	Left colon	47 (32.4)
	Rectum	53 (36.6)
T stage	Tis	2 (1.4)
	T1	3 (2.1)
	T2	6 (4.1)
	T3	89 (61.4)
	T4	45 (31.0)
N stage	N0	35 (24.1)
	N1	47 (32.4)
	N2	63 (43.5)
M stage	M0	83 (57.2)
	M1	62 (42.8)
Stage	0	2 (1.4)
	1	7 (4.8)
	2	17 (11.7)
	3	57 (39.3)
	4	62 (42.8)
Lymphatic invasion	Negative	46 (31.7)
	Positive	81 (55.9)
	Not available	18 (12.4)
Vascular invasion	Negative	80 (55.2)
	Positive	47 (32.4)
	Not available	18 (12.4)
Perineural invasion	Negative	69 (47.6)
	Positive	58 (40.0)
	Not available	18 (12.4)
Differentiation	Well	12 (8.3)
	Moderately	104 (71.7)
	Poorly	15 (10.3)
	Not available	14 (9.7)
Follow-up	Recurrence	37 (25.5)
	Died	5 (3.4)
Chemotherapy	Yes	129 (89.0)
	No	16 (11.0)
Initial CEA^a^	≥ 4	70 (48.3)
	< 4	72 (49.6)
	Not available	3 (2.1)
EGFR immunohistochemistry	0	1 (0.7)
	1 +	54 (37.2)
	2 +	54 (37.2)
	3 +	26 (17.9)
	Not available	10 (7.0)

^a^CEA: carcinoembryonic antigen.

### Mutational profile analysis

**Figure 1:**
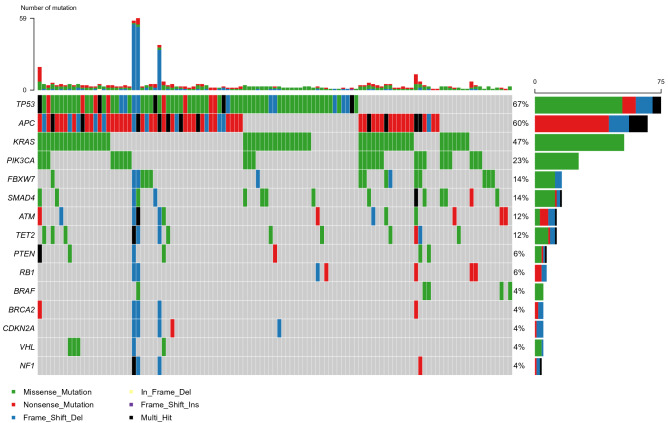
Oncoplot of the top 15 most frequently mutated genes in 115 cases. This shows the list of top 15 genes arranged based on the total number of variants in each gene, and the percentage following each gene represents the ratio of tumor samples with its genetic alteration to the total samples. Colored squares indicate the mutated genes, while gray squares indicate the non-mutated genes. Note 1: The oncoplot was depicted with 115 cases which are available for the genetic alterations of APC. Note 2: Variants annotated as Multi_Hit refer to those genes that mutated more than once in the same sample.

Of total 145 patients, 97.9% (142) showed somatic mutations. Frequent mutations were found in TP53 (70%), APC (60%), KRAS (49%), PIK3CA (23%), FBXW7 (13%), and SMAD4 (12%). The genes with a mutation frequency of > 1% are presented in Fig. 1. In comparison with the mutation frequencies reported by TCGA CRC dataset, the mutation frequencies of TP53, KRAS, and PIK3CA genes were higher in our cohort, whereas APC and FBXW7 mutations were relatively rare^10^ (Fig. A1). In the same manner, the KRAS and PIK3CA mutation rates were higher than the mutation rates reported in the Memorial Sloan Kettering Cancer Center (MSKCC) CRC dataset. On the contrary, our dataset indicated lower rates of APC and SMAD4 mutations^11^ (Fig. A2

**Figure 2:**
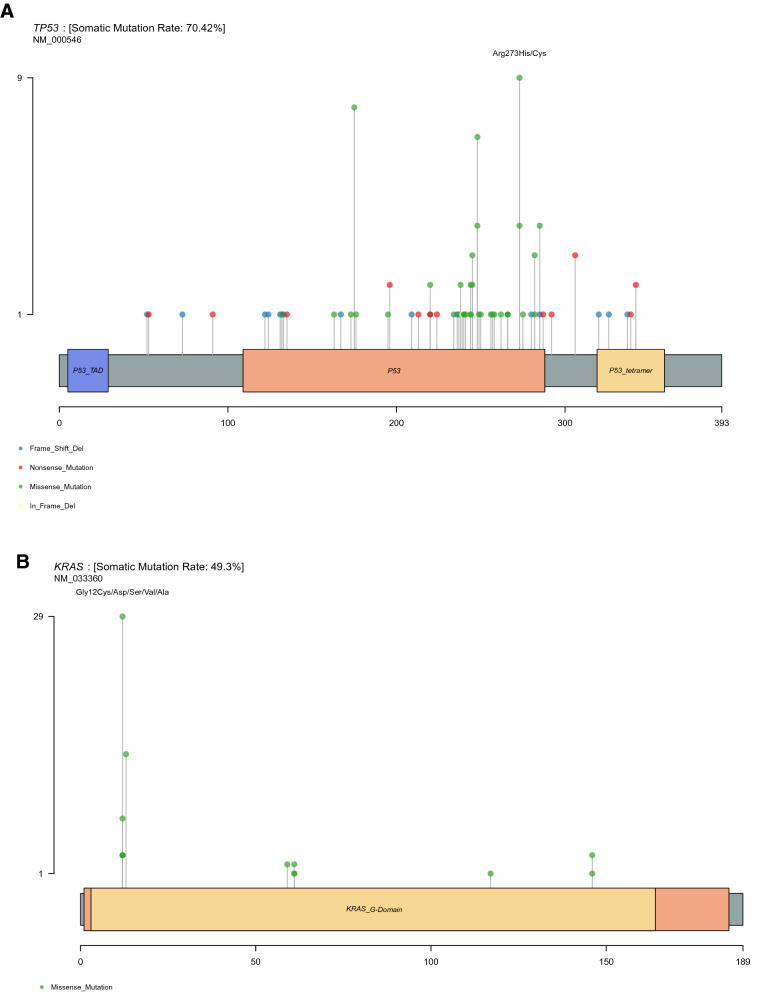

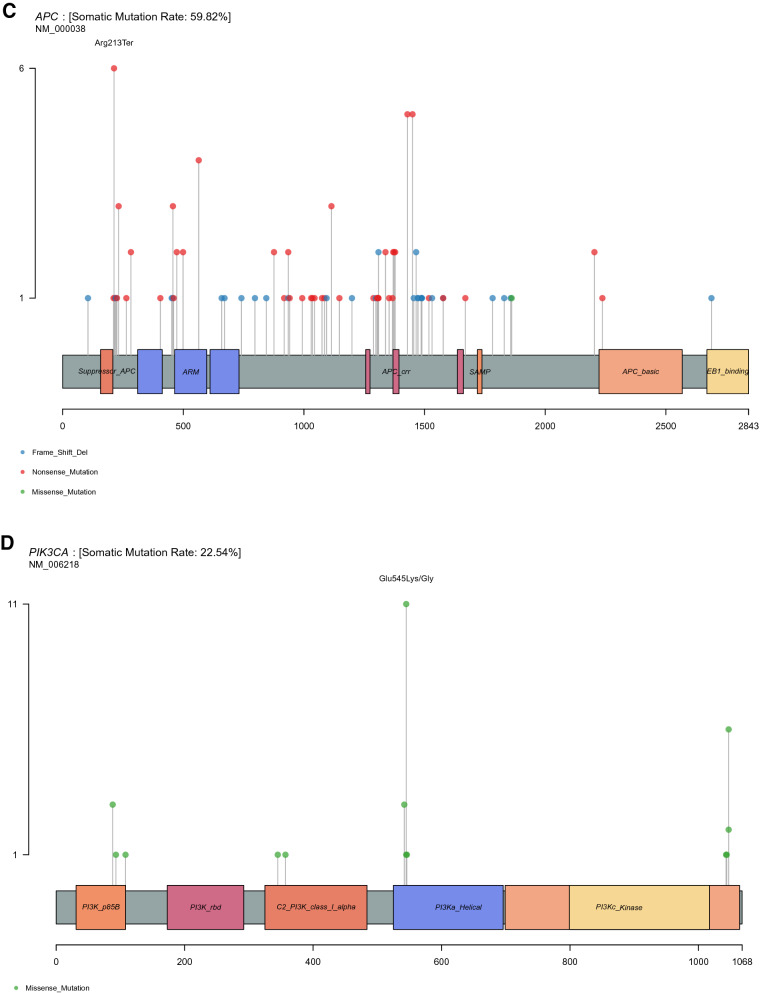

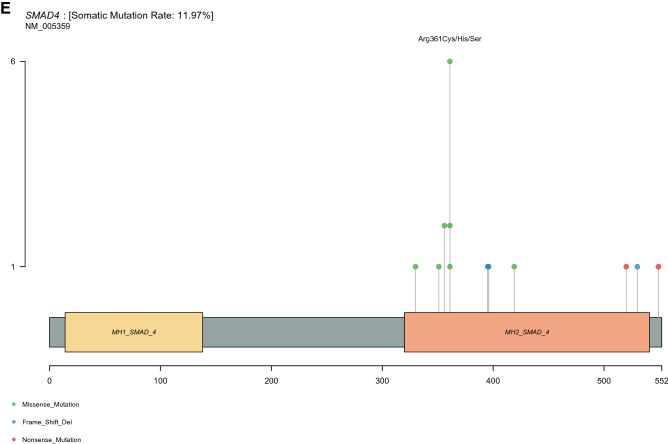
The variant prevalence and spectrum of TP53 (**A**), KRAS (**B**), APC (**C**), PIK3CA (**D**), SMAD4 (**E**) genes in this cohort. All graphs depict a lollipop plot showing identified variants relative to a schematic representation of the gene. Y-axis represent total number of mutations at each residue.

*TP53 mutations.* A total of 107 different TP53 variants were detected from 100 patients. Among pathogenic/likely pathogenic mutations, the most common alterations were those affecting the arginine residue 273 (13 cases; p.Arg273Cys and p.Arg273His) as well as the arginine residues 175 and 248 (8 and 7 cases; p.Arg175His and p.Arg248Trp, respectively) (Fig. 2A and Table A1). In both TCGA and MSKCC CRC datasets, p.Arg175His is the most common variant (15 and 67 cases, respectively) (Figs. A3A, A4A and Table A2, A3, respectively). The presence of missense-type mutant p53 at codon p.Arg175 and p.Arg273 played a key role in the submucosal invasion and metastasis of intestinal tumors through the gain-of-function mechanism^12^.

*KRAS mutations.* Seventy-five mutations from 75 patients were identified in KRAS with the most common affecting glycine 12 residue (45 cases; p.Gly12Asp/Val/Ala/Cys/Ser), followed by changes in the glycine 13 residue (14 cases; p.Gly13Asp) **(**Fig. 2B and Table A1). Likewise, p.Gly12Asp mutation was the most common recurrent variant in both TCGA and MSKCC CRC datasets (Figs. A3B, A4B and Table A2, A3, respectively).

With regard to the overall RAS mutations, one mutation in codon 61 (p.Gln61Lys) of NRAS was also detected. However, no HRAS mutations were reported in our cohort.

*APC mutations.* Because data for APC alterations were not available in 30 cases using Oncomine Comprehensive Assay v3, analysis was performed with 115 case (https://assets.thermofisher.com/TFS-Assets/LSG/brochures/oncomine-comprehensive-assay-v3-flyer.pdf). APC mutations were detected in 67 patients (106 variants). APC mutations were not biased in a particular domain; however, the vast majority of them were truncating mutations. The mutations affecting the arginine 1450 residue, which is one of mutational hotspots for somatic APC mutations, were commonly reported in our study (6 cases; p.Arg213Ter) (Fig. 2C and Table A1)^13^. In TCGA and MSKCC CRC cohorts, the alterations affecting the arginine 1432 (19 cases; p.Arg1432Ter) and 876 residues (44 cases; p.Arg876Ter) were the most common mutations, respectively (Figs. A3C, A4V and Table A2, A3**,** respectively**).**

*PIK3CA mutations.* Thirty-three PIK3CA mutations were detected in 32 patients. The most frequently reported mutations were changes in the glutamic residue 545 in exon 9 of the protein (12 cases; p.Glu545Lys and p.Glu545Gly) (Fig. 2D and Table A1). The mutations affecting the glutamic residue 545 (11 cases; p.Glu545Lys/Ala/Gly/Gln) commonly occurred in the TCGA CRC cohort. Likewise, p.Glu545Lys was the most frequent alteration (52 cases) in the MSKCC CRC dataset (Figs. A3D, A4D and Table A2, A3, respectively**).**

*SMAD4 mutations.* Nineteen mutations from seventeen patients were identified in the SMAD4 genes. Of these, pathogenic/likely pathogenic mutations were detected in 11 variants with the most common affecting arginine residue 361, which is the hotspot region for missense mutations (6 cases; p.Arg361His) (Fig. 2E and Table A1)^14^. In the same manner, the most frequent variant was also detected at arginine residue 361 in TCGA (8 cases; p.Arg361His) and MSKCC CRC datasets (32 cases; p.Arg361His and p.Arg361Cys) (Figs. A3E, A4E and Table A2, A3, respectively).

The remaining recurrent mutations identified are summarized in Table A1**.**

**Figure 3:**
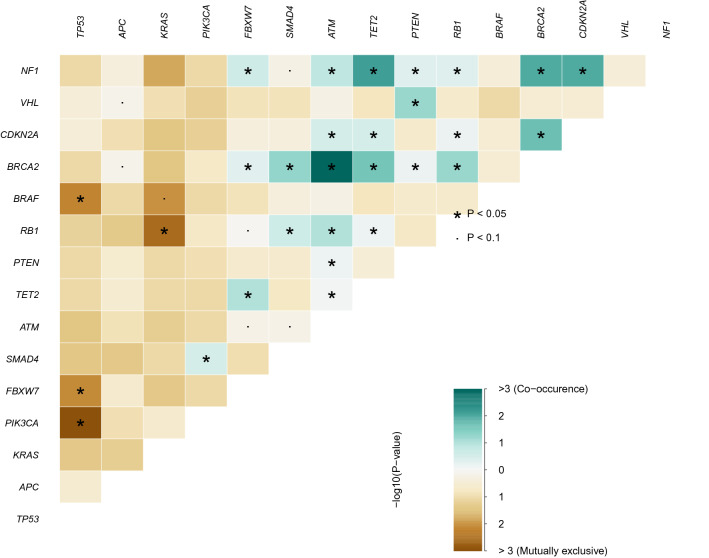
Mutually exclusive and co-occurring gene pairs in our dataset presented in a triangular matrix. Bluish green indicates a tendency toward co-occurrence, whereas brown indicates a tendency toward mutually exclusiveness.

In the present study, the presence of TP53 mutations showed mutual exclusivity with that of PIK3CA mutations, which is also shown in both TCGA and MSKCC cohorts. These findings are in line with that evidence that PI3CA and TP53 alterations tend to be mutually exclusive in diverse tumors^15^. Likewise, TP53 and FBXW7 alterations was mutually exclusive in our cohort. TP53 and BRAF alterations also showed mutually exclusiveness. On the contrary, the ATM mutations positively correlated with BRCA2 mutations, which was also confirmed with the MSKCC CRC data. Mutually exclusive or co-occurring set of genes was detected in our dataset, and TCGA and MSKCC cohorts are shown in Fig. 3, and A5 and A6, respectively.

### Outcome analysis

We evaluated the prognostic impact of the mutational profile on clinical outcomes. TP53 mutations were significantly associated with lymph node metastasis (p = 0.01803) and higher overall stage (p = 0.03813). BRAF mutation was predominantly harbored by the presence of metastasis (0.04222). In addition, FBXW7 and ATM mutations were significantly associated with higher tumor stage (p = 0.03191 and 0.01237, respectively). The association of common gene mutations in CRC with TNM classification and overall stage are summarized in Table 2. In terms of the correlation between gene mutation and demographic and pathological parameters, the frequency of mutations affecting FBXW7 was higher in male patients (p = 0.01063) (Table 2).

**Table 2:** Correlation of common gene mutations in CRC according to the clinicopathological data of our cohort.

Mutations	T stage		N stage		M stage		Stage					
	1, 2	3, 4	0	1, 2	0	1	1, 2	3, 4				
**RAS**	p = 0.8676		p = 0.4023		p = 0.5007		p = 0.7053					
Negative	6	63	14	55	42	27	11	58				
Positive	5	71	21	55	41	35	15	61				
**BRAF**	p = 1		p = 0.6752		**p = 0.04222**		p = 1					
Negative	11	127	33	105	82	56	25	113				
Positive	0	7	2	5	1	6	1	6				
**PIK3CA**	p = 0.7073		p = 0.7166		p = 0.05119		p = 0.9012					
Negative	8	105	26	87	70	43	21	92				
Positive	3	29	9	23	13	19	5	27				
**TP53**	p = 0.09612		**p = 0.01803**		p = 0.4125		**p = 0.03813**					
Negative	6	39	17	28	23	22	13	32				
Positive	5	95	18	82	60	40	13	87				
**APC**^c^	p = 0.7598		p = 1		p = 0.2234		p = 0.9395					
Negative	4	44	10	38	30	18	9	39				
Positive	7	60	15	52	33	34	11	56				
**SMAD4**	p = 1		p = 1		p = 0.904		p = 0.5087					
Negative	10	118	31	97	74	54	22	106				
Positive	1	16	4	13	9	8	4	13				
**FBXW7**	**p = 0.03191**		p = 0.1424		p = 0.9203		p = 0.09594					
Negative	7	120	28	99	72	55	20	107				
Positive	4	14	7	11	11	7	6	12				
**ATM**	**p = 0.01237**		p = 0.7439		p = 1		p = 0.717					
Negative	7	124	31	100	75	56	23	108				
Positive	4	10	4	10	8	6	3	11				

Mutations	Age		Sex		Differentiation^a^		Lymphatic invasion^b^		Vascular invasion^b^		Perineural invasion^b^	
	< 60	≥ 60	M	F	W to M	Poorly	Absent	Present	Absent	Present	Absent	Present
**RAS**	p = 0.8369		p = 0.437		p = 0.9249		p = 0.1176		p = 0.9134		p = 0.972	
Negative	33	36	39	30	56	8	17	43	37	23	32	28
Positive	34	42	37	39	60	7	29	38	43	24	37	30
**BRAF**	p = 0.2491		p = 0.2577		p = 0.1002		p = 1		p = 1		p = 0.3746	
Negative	62	76	74	64	113	13	44	78	77	45	65	57
Positive	5	2	2	5	3	2	2	3	3	2	4	1
**PIK3CA**	p = 0.6054		p = 0.3622		p = 0.3053		p = 1		p = 0.8195		p = 0.7176	
Negative	54	59	62	51	90	14	36	64	64	36	53	47
Positive	13	19	14	18	26	1	10	17	16	11	16	11
**TP53**	p = 1		p = 0.6962		p = 0.1422		p = 0.08001		p = 0.2426		p = 0.201	
Negative	21	24	22	23	32	7	19	20	28	11	25	14
Positive	46	54	54	46	84	8	27	61	52	36	44	44
**APC**^**c**^	p = 0.8859		p = 0.6938		p = 0.09394		p = 0.5736		p = 0.1451		p = 0.9066	
Negative	23	25	27	21	37	9	16	27	29	14	22	21
Positive	30	37	34	33	55	4	17	40	29	28	31	26
**SMAD4**	p = 0.8541		p = 0.832		p = 0.6913		p = 0.3429		p = 1		p = 0.9175	
Negative	60	68	68	60	100	14	38	73	70	41	61	50
Positive	7	10	8	9	16	1	8	8	10	6	8	8
**FBXW7**	p = 1		**p = 0.01063**		p = 1		p = 0.4667		p = 0.6693		p = 0.2363	
Negative	59	68	61	66	101	13	38	72	68	42	57	53
Positive	8	10	15	3	15	2	8	9	12	5	12	5
**ATM**	p = 0.986		p = 0.5129		p = 0.6284		p = 0.35		p = 0.7595		p = 0.5505	
Negative	60	71	67	64	106	13	40	75	73	42	61	54
Positive	7	7	9	5	10	2	6	6	7	5	8	4

^a^A total of 14 cases have no available data.

^b^A total of 18 cases have no available data.

^c^A total of 30 cases have no available data.

For TCGA dataset, TP53 and BRAF mutations were significantly associated with lymph node metastasis (p = 0.00025 and 0.00398, respectively) and higher overall stage (p = 0.00016 and p = 0.00195, respectively), which are consistent with our cohort. Furthermore, PIK3CA mutations correlated with lower N stage and overall stage (p = 0.0217 and 0.00253, respectively). In addition, ATM mutations were significantly associated with the absence of metastasis (p = 0.00766) (Table A4).

**Figure 4:**
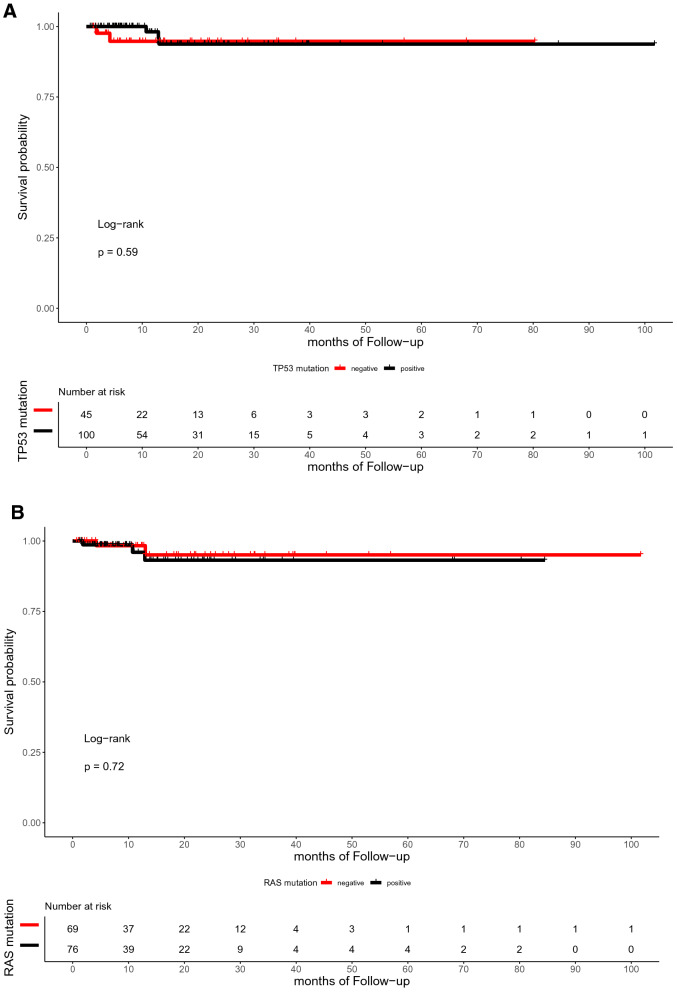

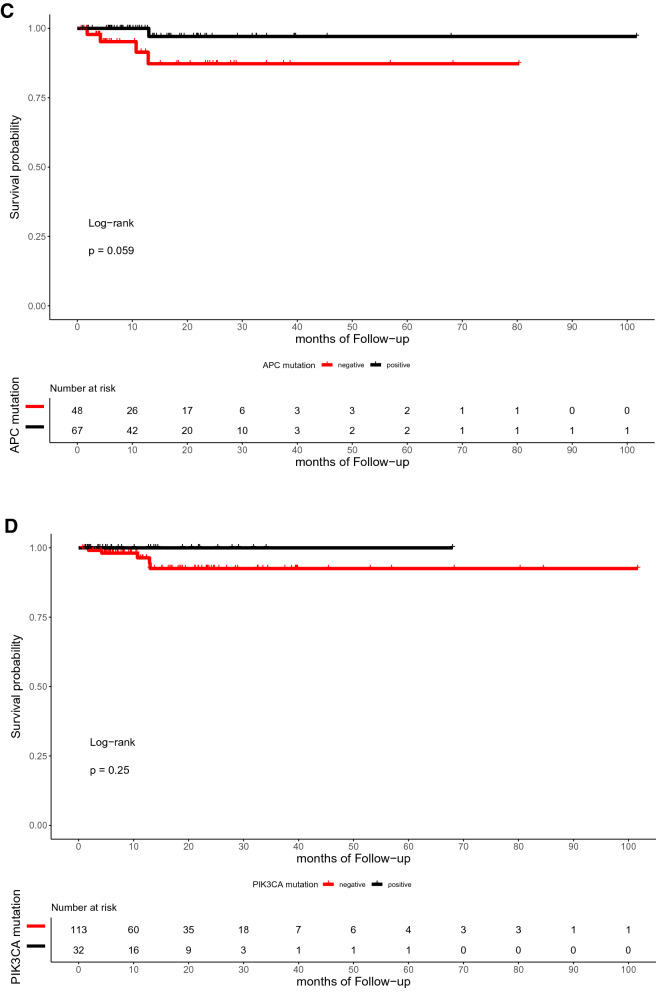

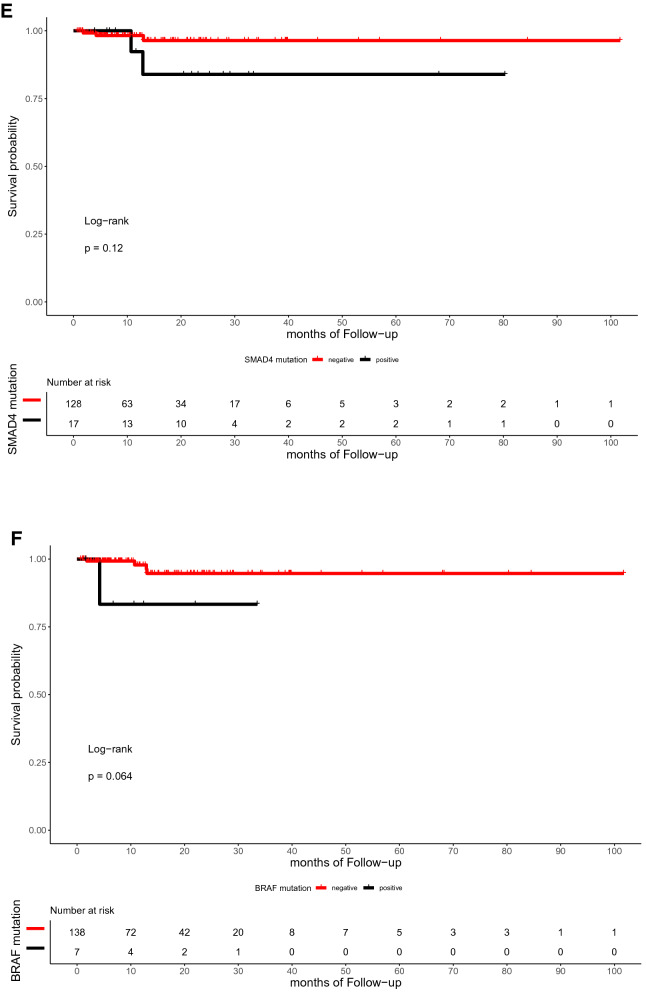

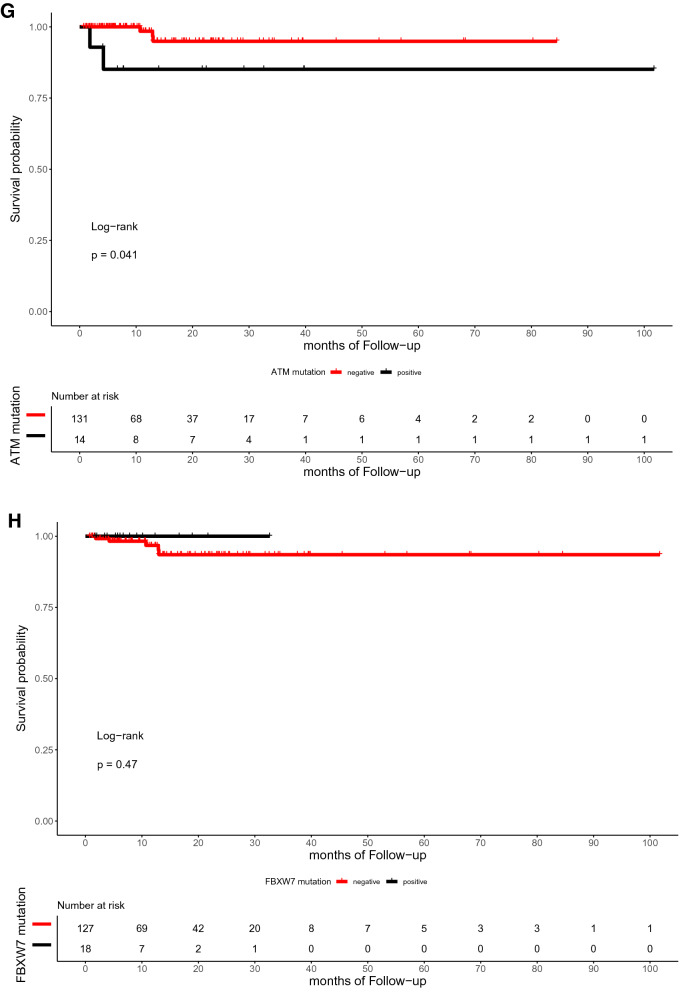
Kaplan–Meier curve for OS in 145 CRC patients by mutational status, including TP53 (**A**), RAS (**B**), APC (**C**), PIK3CA (**D**), SMAD4 (**E**), BRAF (**F**), ATM (**G**), and FBXW7 (H).

**Figure 5:**
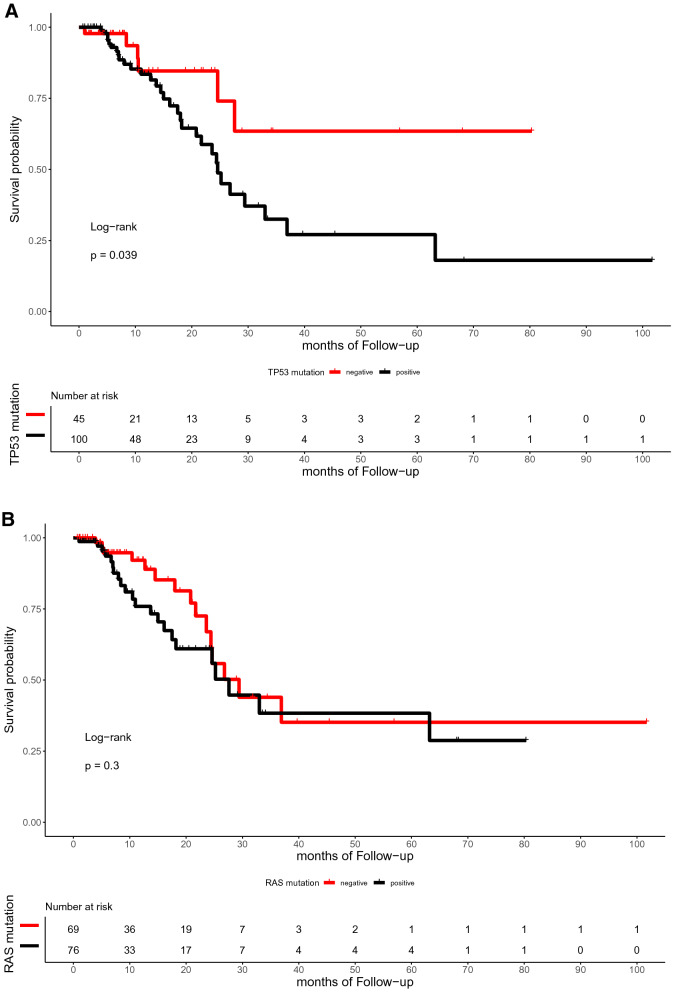

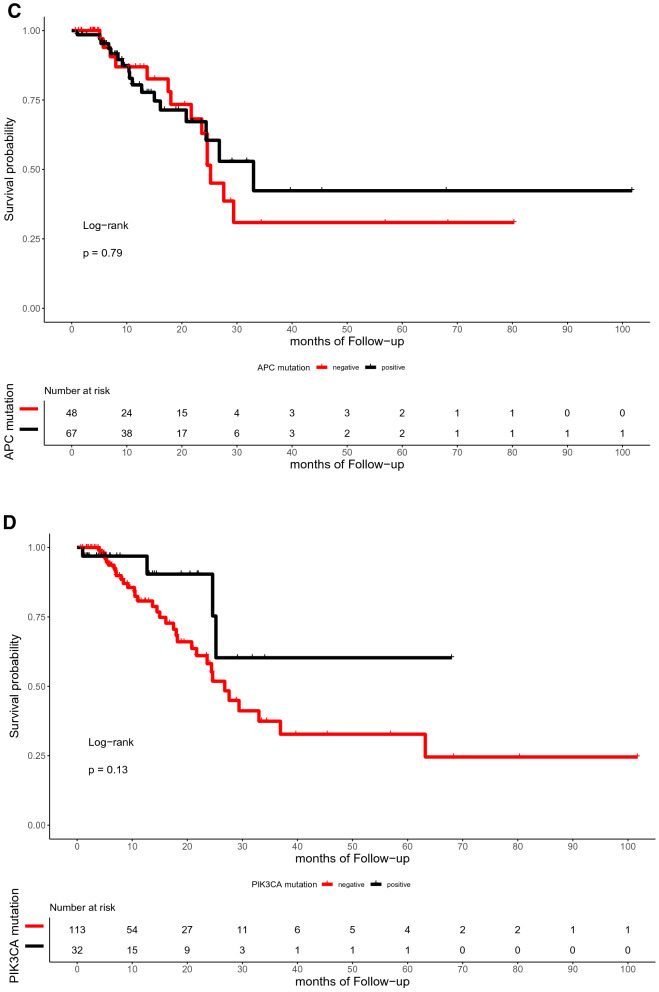

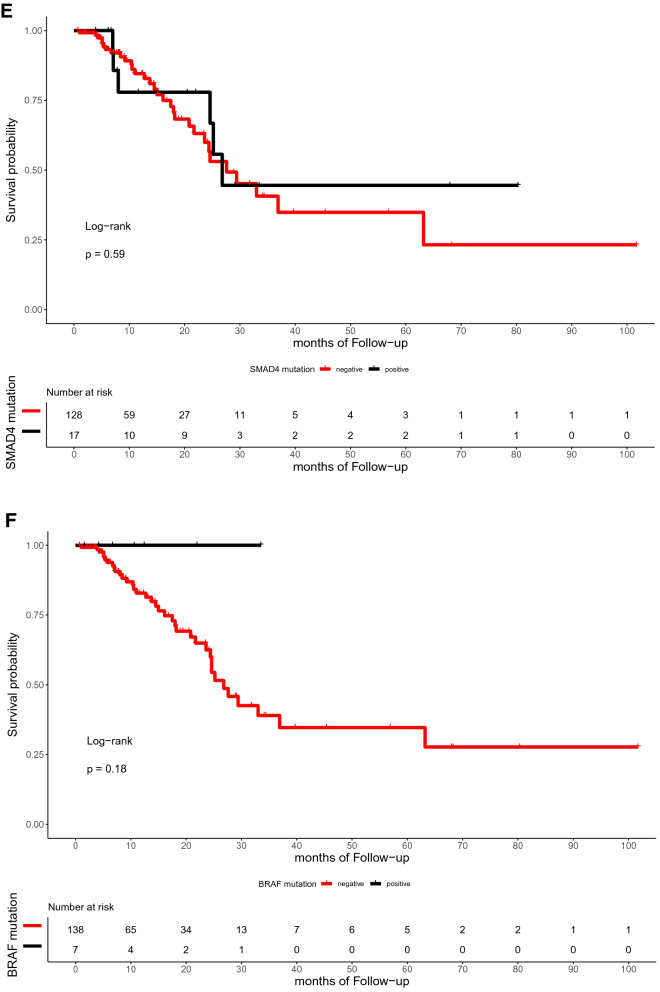

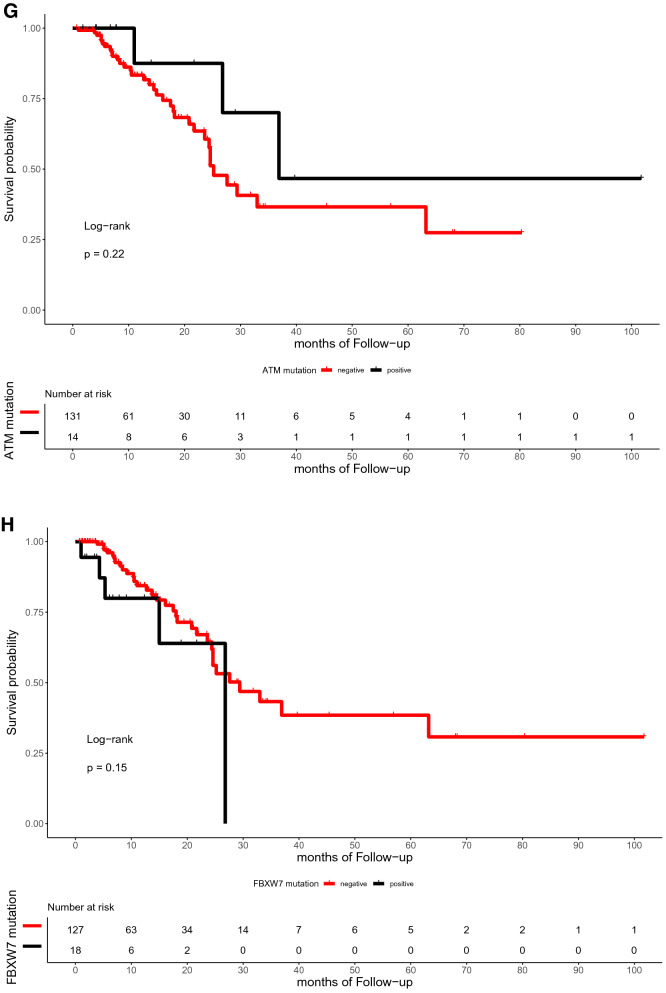
Kaplan–Meier curve for DFS in 145 CRC patients by mutational status, including TP53 (**A**), RAS (**B**), APC (**C**), PIK3CA (**D**), SMAD4 (**E**), BRAF (**F**), ATM (**G**), and FBXW7 (**H**).

The median time of follow-up was 16.5 months (range, 0.7–101.7 months). In the present study, the OS of patients with or without TP53, RAS, APC, PIK3CA, and SMAD4 mutations did not differ significantly (p = 0.59, 0.72, 0.059, 0.25, and 0.12, respectively; Fig. 4). Similarly, the DFS between patients with RAS, APC, PIK3CA, and SMAD4 mutations and those with wild-type were not statistically different (p = 0.3, 0.79, 0.13, and 0.59, respectively; Fig. 5). However, the DFS of patients with TP53 mutation was significantly shorter than that of patients with TP53 wild type (p = 0.039; Fig. 5A). Furthermore, ATM mutation was significantly associated with shorter OS (p = 0.041, Fig. 4G).

With regard to the subset analysis of stage 3 and 4 patients, those with ATM mutation showed shorter OS (p = 0.023, Fig. A7A). BRAF mutation showed significant correlations with shorter OS (p = 0.042, Fig. A7B), which was not observed among the entire study population. The FBXW7 mutation was significantly associated with shorter DFS in patients with stage 3 and 4 CRC (p = 0.002, Fig. A8A). There was a trend toward a poor DFS for CRC in the group with TP53 mutation, although this trend was not statistically significant (p = 0.073, Fig. A8B).

For the TCGA CRC dataset, the OS was not significantly different between patients with TP53, KRAS, APC, PIK3CA, SMAD4, and NRAS mutations and their wild types (p = 0.58, 0.96, 0.89, 0.38, 0.19, and 0.99, respectively; Figure A9). For the MSKCC CRC cohort, there was no difference in OS between patients with TP53, KRAS, PIK3CA, SMAD4, and NRAS mutations and wild type (p = 0.42, 0.39, 0.4, 0.26, and 0.16, respectively; Figure A10). On the contrary, BRAF mutation was associated with shorter OS, compared with wild-type mutation (p < 0.0001; Figure A10F). The absence of APC was correlated with poor clinical outcomes (p = 0.012; Figure A10C). Contrary to our results, the mutational statuses of ATM and FBXW7 did not differ significantly in terms of OS (p = 0.34 and 0.14, respectively; Figure A10H and A10I).

**Figure 6:**
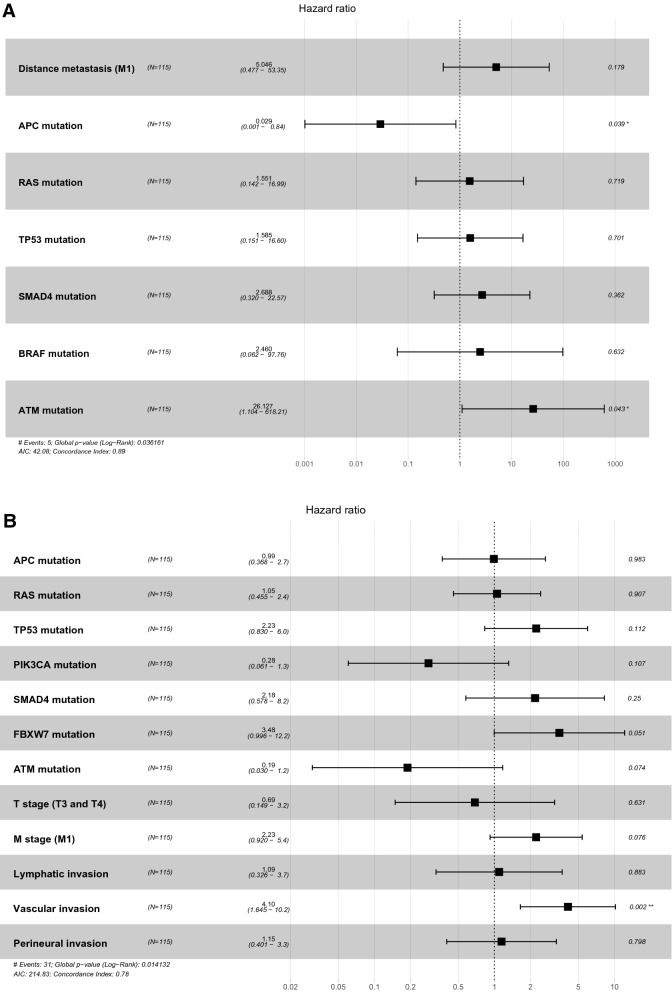
Forest plots for CRC prognostic markers. Multivariable Cox-proportional hazards analysis was performed to identify the prognostic markers that may predict OS (**A**) and DFS (**B**). Error bars represent hazard ratios and their 95% confidence intervals.

In our cohort, the multivariate Cox’s regression analysis of OS showed that ATM mutation is an independent biomarker for poor prognosis with a hazard ratio of 19.637 (p = 0.043, confidence interval [CI] = 1.104–618.21). In the same manner, TP53 and FBXW7 mutations were poor prognostic factors for DFS with hazard ratios of 2.23 (p = 0.112, CI = 0.830–6.0) and 3.48 (p = 0.051, CI = 0.996–12.2), respectively. Figure 6A and B summarizes the role of clinicopathologic parameters and individual mutations on OS and DFS, respectively.

Interestingly, the hazard ratio of APC mutation was low (0.029; p = 0.039), which is different from other genetic mutations. Previous study demonstrated that CRCs lacking any APC mutation resulted in a worse prognosis than tumors with single APC truncating mutation. Among 115 cases available for APC mutational status in our cohort, there were 39 cases of single mutation, with 48 cases of non-mutation and 28 cases of multi-mutations in APC. In addition, although APC is quite frequently mutated, known driver gene in CRC, it has not generally been included as a prognostic factor in clinical setting^16^. Therefore, for the reasons described above, it is thought that the hazard ratio of APC mutations was relatively lower in this study.

### Variant allele frequency analysis

Based on the assumption that VAF is a potential biomarker^17^, we investigated the biological and clinical significance of VAF for small variants (SNVs, DNVs, and indels) in our cohort as well as in the public datasets (TCGA and MSKCC cohorts). Among clinically actionable mutations, TP53 showed the highest VAF (Figure A11), and higher VAFs were significantly correlated with higher stages in APC and SMAD4 mutations (p = 0.044 and 0.047, respectively). The distribution of median VAFs in our cohort were extremely left shifted (Figure A12). These trends were similar to those in the MSKCC cohort, but significantly different from those in TCGA CRC cohort (p < 0.001), indicating the higher sensitivity of targeted deep sequencing over whole exome sequencing. Among clinically significant small variants in our cohort, the proportions of VAF less than 0.15 and 0.1 were 39.3% and 29.7%, respectively, suggesting that a low VAF variant should not be neglected.

**Figure 7:**
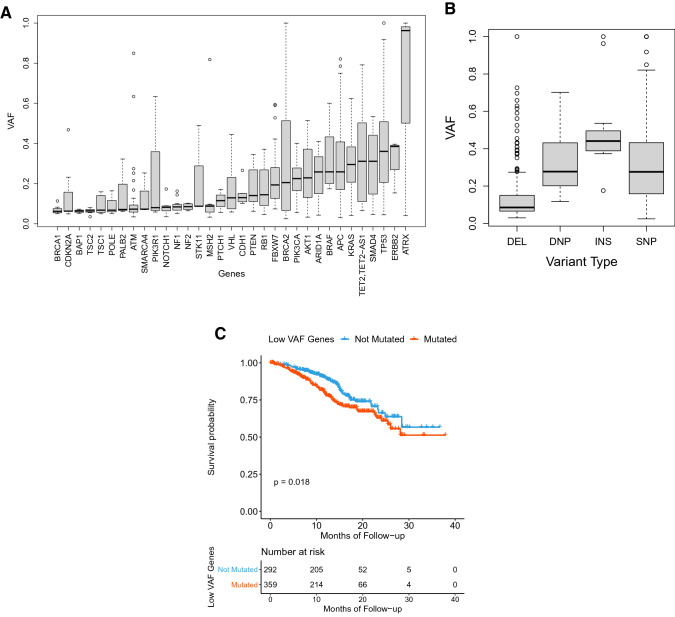
Gene-wise distribution of variant allele frequencies (VAF) for genes with more than two instances of somatic mutations (**A**), distribution of VAFs depending on mutation types (**B**), and Kaplan–Meier curve for OS in the MSKCC cohort stratified by presence of genes with relatively lower VAF (**C**).

We investigated whether certain genes are more likely to have either lower or higher VAF when mutated. When only 34 VAF-comparable genes (genes with more than two instances of VAFs) were considered, the gene-specific distribution of VAF were easily identified in spite of the large variability (Fig. 7A). These trends were relatively preserved in the MSKCC cohort. Gene-wise median VAFs were weakly associated with gene-wise instances of VAFs, implying that more frequently mutated genes were more likely to have higher VAFs (Figure A13A). These findings straightforwardly suggest that the variations in VAF cannot be entirely explained by technical reasons, and some of the biological features of genes may be associated with VAF. To better understand VAF in relation to mutation type, we explored the distributions of VAF based on the type of mutation (Fig. 7B**).** Although VAF seems significantly different in terms of mutation type, these trends were not observed in the MSKCC cohort (Figure A13B). These differences might be due to the variations in the NGS platform rather than in cancer biology.

For the evaluation of clinical relevance of low VAF variants, we classified the 34 genes into low VAF genes and high VAF genes by sorting them according to their median VAFs (Table A5). Each of the clinical cases was classified into “low VAF gene predominant” and “high VAF gene predominant” depending on which type of genes is predominant among the list of mutated genes in each case. No significant difference was shown in OS and DFS between the subgroup with high VAF variants and that with low VAF variants (Figure not shown).

### Comparison of clinicopathological parameters between MSI and MSS CRCs

Of 145 tumors, 133 were included in the MSI analysis. The correlations between MSI status and the clinicopathologic features as well as the mutations of major driver genes in CRC are summarized in Table 3. Microsatellite instability-high (MSI-H) CRCs revealed a marked predilection for the right colon (p = 0.009651). MSI-H tumors showed lower T, N, and overall stages than MSI-L/MSS tumors (p = 0.004249, 0.002777, and 0.003935, respectively). However, no significant differences were observed in the M stage between patients with MSI-H tumors and those with MSI-L/MSS tumors (p = 0.08775). For genetic alterations, MSI-H tumors were characterized by infrequent KRAS mutation (p = 0.04454). By contrast, no significant association was found between MSI status and PIK3CA or BRAF mutation (p = 0.6935 and 0.3277, respectively). For the TCGA cohort, patients with PIK3CA and BRAF mutations were deviated in the MSI-H groups (p = 0.03334 and < 0.001, respectively, Table A6).

**Table 3:** Correlation of MSI status according to the clinicopathological data and RAS, PIK3CA, and BRAF mutations in our cohort.

Clinicopathological parameters	MSI status^a^	
	MSS/MSI-L	MSI-H
**Age**	p = 0.1853	
≥ 60	54 (43.9%)	7 (70.0%)
< 60	69 (56.1%)	3 (30.0%)
**Sex**	p = 0.7448	
Male	62 (50.4%)	6 (60.0%)
Female	61 (49.6%)	4 (40.0%)
**Tumor site**	**p = 0.009651**	
Right colon	34 (27.6%)	7 (70.0%)
Left colon	89 (72.4%)	3 (30.0%)
**T stage**	**p = 0.004249**	
1, 2	7 (5.7%)	4 (40.0%)
3, 4	116 (94.3%)	6 (60.0%)
**N stage**	**p = 0.002777**	
0	27 (22.0%)	7 (70.0%)
1, 2	96 (78.0%)	3 (30.0%)
**M stage**	p = 0.08775	
0	72 (58.5%)	9 (90.0%)
1	51 (41.5%)	1 (10.0%)
**Stage**	**p = 0.003935**	
1, 2	20 (16.3%)	6 (60.0%)
3, 4	103 (83.7%)	4 (40.0%)
**Differentiation**^**b**^	p = 1	
Well/ Moderately	102 (12.1%)	9 (90.0%)
Poorly	14 (87.5%)	1 (10.0%)
**Lymphatic invasion**^**c**^	p = 0.7459	
Absent	41 (35.7%)	4 (40.0%)
Present	74 (64.3%)	6 (60.0%)
**Vascular invasion**^**c**^	p = 0.09041	
Absent	70 (60.9%)	9 (90.0%)
Present	45 (39.1%)	1 (10.0%)
**Perineural invasion**^**c**^	**p = 0.02117**	
Absent	59 (51.3%)	9 (90.0%)
Present	56 (48.7%)	1 (10.0%)
**Initial CEA level (blood)**^**d**^	**p = 0.01908**	
≥ 4	60 (50.0%)	9 (90.0%)
< 4	60 (50.0%)	1 (10.0%)
**RAS mutation**	**p = 0.04454**	
Absent	54 (43.9%)	8 (80.0%)
Present	69 (56.1%)	2 (20.0%)
**PIK3CA mutation**	p = 0.6935	
Absent	96 (78.0%)	7 (70.0%)
Present	27 (22.0%)	3 (30.0%)
**BRAF mutation**	p = 0.3277	
Absent	119 (96.7%)	9 (90.0%)
Present	4 (3.3%)	1 (10.0%)

^a^A total of 12 patients have no MSI test result.

^b^A total of 14 patients have no available data .

^c^A total of 18 patients have no available data .

^d^Three patients have no available data.

Among the patients with MSI-L/MSS tumors, the OS and DFS was not significantly different between patients with overall stage 1 & 2 vs 3 & 4 (p = 0.3 and 1, respectively; Figure A14A and A14B). Likewise, the OS and DFS did not differ significantly between patients with M stage 0 vs 1 (p = 0.12 and 0.42, respectively; Figure A15A and A15B). For the TCGA CRC dataset, however, higher stage was associated with shorter OS, compared with lower stage (p < 0.0001; Figure A16A). In the same manner, distant metastasis was correlated with shorter OS (p < 0.0001; Figure A16B). The proportion of stage 3 and 4 in our dataset was much higher than that of TCGA dataset (p < 0.001), which may affect the conflicting result between two cohorts.

Of ten cases with MSI-H group, seven patients showed loss of expression in MLH1 protein. MSH2 was revealed in one patient. In one case, BRAF mutation was found, with loss of MSH6 protein expression. The detailed information for MSI-H group is summarized in Table A7.

### CNA and fusion gene analysis

Before conducting the main analysis, as a sanity check, we investigated whether the degree of copy number change in a case is correlated with a particular gene involved. The copy numbers of sufficiently remote gene pairs would not be correlated without the occurrence of rare events such as whole genome duplications. When gene pairs were randomly chosen in cases with more than two copy number changes, the correlation of copy numbers was higher than expected when a permutation test was performed by permuting the case labels and then the distribution of the Spearman’s correlation of copy numbers was tested (p = 0.022). These results indicate that the suggested copy numbers of each gene should be carefully interpreted, and the possibility of some technical artifacts should be considered.

**Figure 8:**
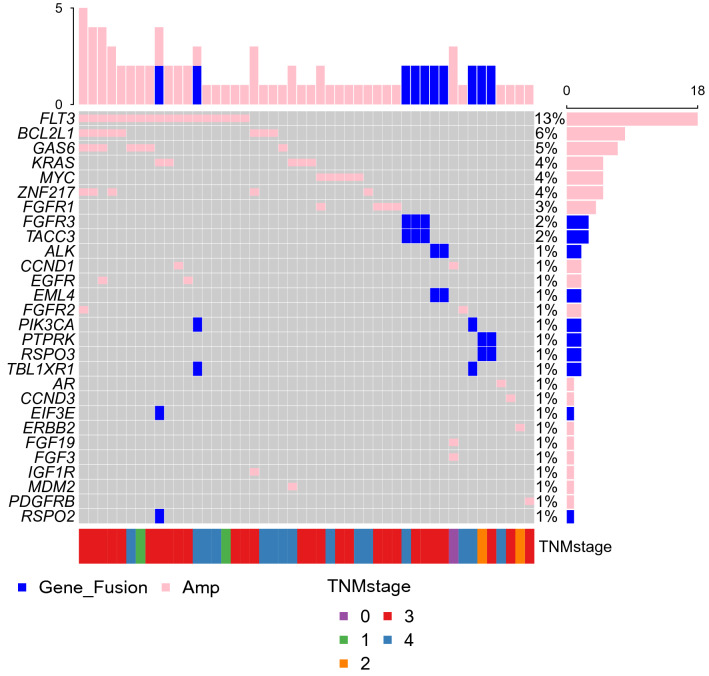
Oncoplot of all genes in which either gene fusion or copy number variation (CNV) was identified. This figure shows the list of genes arranged based on the total number of variants in each gene and the percentage of each gene, which is the ratio of tumor samples with this genetic alteration to total samples.

Because the copy number itself may be less accurate, but amplification or deletion, whose property is different from the copy number, is less dependent on the scaling factor, the main analysis was performed only with the occurrence of either amplification or deletion. Copy number aberrations were detected in 38 of 139 samples (27.3%), all of which being amplification (Fig. 8). A total of 18 genes were amplified with FLT3 (being the most frequent) and BCL2L1, GAS6, KRAS, MYC, ZNF217, and FGFR1 (being recurrent). All of these genes are well-known oncogenes, and most of them are targetable with specific inhibitors.

Fusion genes were identified in 10 cases, and a total of 10 genes were involved, with FGFR3 as the most frequent gene. At least one of the fusion partners were well-known oncogenes in all cases.

## Discussion

In this study, we showed that TP53 is the most commonly mutated gene in CRC, followed by KRAS and APC. However, APC mutation, followed by TP53 and KRAS mutations, is mostly common in both TCGA and MSKCC CRC datasets. The rates of TP53 mutation are similar to those reported in our cohort and MSKCC dataset (70% vs 73%, respectively); however, the rate of TP53 mutation reported in the TCGA CRC dataset is lower (54%). The frequency of APC mutations reported in this study was 60%, which is significantly lower than that reported in the TCGA and MSKCC studies (71% and 76%, respectively) (Fig. 1 and A1 and A2). This difference in mutational frequency as well as distribution in codons among our study, TCGA and MSKCC CRC, may be attributable to the differences in the sample selection, ethnicity, and methodology for genomic studies.

For APC gene, the targeted NGS panel (Oncomine Comprehensive Assay v1) detects most of the exons. However, it is designed not to call the variants in some ‘masking areas’ where the mutation detected may be highly false positive. In addition, it does not cover introns. Moreover, there is a potential association of a lack of APC mutations with respect to the early onset CRC phenotype^18,19^. The mean age in our study cohort was significantly lower than that of TCGA CRC dataset, (60.9 ± 13.18 vs 65.1 ± 13.0, p < 0.001), which might partly reflect the difference in APC-mutation frequency between cohorts. Unfortunately, the MSKCC dataset had no information about patient age.

A recent Chinese study on CRC using whole exome sequencing (WES) revealed mutation rate of 59.38% in APC, which is similar to our study result^20^. In addition, the mutation rate TP53 is 50%, which is lower than that of our cohort. In another study from Saudi Arabia, the targeted sequencing identified 36% of mutations in APC, which is significantly lower than our cohort TCGA and MSKCC dataset^21^. However, the Cancer Hotspot Panel 2.0 was used in their study, which detects only hot spot mutations of APC gene. TP53 mutation rate was 65%, which is slightly lower than our study result.

KRAS mutated in 49% of CRC patients, which is in accordance with the frequencies reported in various studies on CRCs (35%–45%) including TCGA and MSKCC datasets (42% and 44%, respectively)^22,23^. The presence of KRAS mutations is considered as predictive markers of negative pharmacological response to EGFR inhibitors, such as cetuximab or panitumumab^24,25^. However, the prognostic role of KRAS mutations for DFS and OS in CRC patients remains controversial. Various retrospective studies did not show any prognostic significance, but some confirmed that KRAS mutations had negative prognostic role for DFS, OS, or liver metastases^26–28^. In our study, we did not indicate the relationship between RAS mutation and the clinicopathological parameters.

In our study, the BRAF mutation frequency was 4.8% (7/145), which was lower than that identified in the Western cohort (9.2%) but similar to the mutation rates identified in Asian countries (4.9%)^23^. Previous studies have widely demonstrated that patients with CRC who possess a BRAF mutation have significantly poorer clinical outcomes^4,29^. This was observed also in the present study; BRAF mutations were not only associated with metastatic tumors, but also found to be a negative prognostic marker for OS in a subgroup analysis with advanced CRCs.

Besides the frequently mutated actionable genes, ATM and FBXW7 mutations tended to be a poor prognostic factor in the multivariate analyses of OS and DFS, respectively.

ATM is a member of the phosphatidylinositol-3 kinase-like family of serine/threonine protein kinases and plays a pivotal role in the cellular response to DNA damage by ionizing radiation, which results in DNA double-strand breaks^30^. With regard to the ATM gene alterations, 10% of our cohorts carried mutations. The TCGA and MSKCC dataset reported that ATM mutations were detected in 14% and 7% of CRC patients, respectively. Randon et al. found that ATM mutations are independently associated with longer OS in patients with metastatic CRC^31^. However, we revealed that ATM mutation is linked to poorer OS in patients with CRC. Furthermore, the subgroup analysis of stage 3 and 4 CRCs also identified that ATM mutation is significantly associated with shorter OS. In line with this, loss of ATM expression showed a tendency toward worse survival rate in patients with CIN CRC^32^. In addition, no differences were observed in the OS according to ATM mutation status in both TCGA and MSKCC cohorts. The discrepancy among studies might be due to the different prognostic role of ATM according to the disease stage and the confounding factors from the heterogeneity of available treatment strategies used for metastatic disease^31^.

The frequency of FBXW7 mutation in the present study was 13%. This value is consistent with that in previous studies, which reported that 10% of patients with CRC have FBXW7 mutations^33^. FBXW7 is a potential tumor suppressor, and mutations in the gene are thought to impair cyclin E degradation resulting in uncontrolled cell division and growth, thus resulting in cancer progression^34^. A previous study suggested that a missense mutation was correlated with poor OS in CRC patients^35^. We revealed that FBXW7 mutations correlated with shorter DFS in the subgroup with advanced cancer stage.

SMAD4 plays an essential role as mediator in the transforming growth factor-β pathway. Sporadic mutations of SMAD4 are identified in up to 20.0% of CRCs^36^. In this study, SMAD4 mutation was present in 14% of cases. While previous study reported that SMAD4 mutation was associated shorter overall survival than in wild-type SMAD4 cases^36^, this study revealed that SMAD4 mutation was not associated with poor clinical outcome, which is the same in both TCGA and MSKCC cohorts.

For TET2, PTEN, and BRCA2 alteration, the mutational frequencies ranked within top 12 in our study result. However, these mutations were relatively rare in both TCGA and MSKCC datasets. The role of BRCA2 in CRC needs yet to be elucidated. Based on the current literature BRCA2 deficiencies are not considered traditionally associated with CRC and do not confer in disease establishment.

MSI status is considered as an independent prognostic indicator. In our study, MSI-H CRCs showed significant association with lower T stage, N stage, and clinical stage. Likewise, previous studies generally revealed a better clinical outcome in patients with MSI-H CRCs compared with those with MSS tumors^37,38^. In our cohort, KRAS-mutated tumors were more frequently found in the MSS group, which is consistent with the findings of previous studies^29,39^. A previous study by Koyel et al. reported a strong association between high CEA (≥ 4) and MSI-H, which is also reflected in the present study^40^.

Of ten patients in MSI-H group, seven patients (case 2, 4, 5, 6, 8, 9 and 10) showed negative staining in MLH1 protein (Table A7). A considerable proportion of MSI-H CRCs observed in non–Lynch syndrome results from hypermethylation of the MLH1 promoter without the gene mutation^41^. For case 2, MSH2 mutation might be considered as non-functional alteration, based on proficient MSH2 expression. For case 6, additional study is needed to determine whether the MLH1 mutation is somatic or germline.

Interestingly, case 1 showed negative staining for MSH6 and BRAF D594N mutation (Table A7). It is known that BRAF V600E mutation hardly ever occurs in MSI-H CRCs due to Lynch syndrome. Hence, BRAF V600E mutation is used as a practical marker for hypermethylation in CRCs with loss of MLH1 expression^42^. However, researches on the relationship between BRAF non‐V600E mutations and MSI status remain scarce, thus, further studies are needed for determining the clinical significance of MSI-H tumors with BRAF non‐V600E mutations^43^. Moreover, researchers have shown that loss of PMS2 expression and MLH1 proficient CRCs or tumors with MSH2 and/or MSH6 negativity are highly associated with Lynch syndrome^44^. Therefore, case 1 cannot rule out the possibility of Lynch syndrome, however, germline mutation test is required for confirmation.

In terms of cancer evolution, the driver mutations are likely to have a relatively high VAF due to their emergence in the earlier stage of carcinogenesis. On the contrary, actionable variants induced by a treatment may develop with a low VAF in patients with advanced cancer^17,45^. Recently, the clinical relevance of low VAF variants on the survival of patients who received a targeted therapy was suggested. If the low VAF mutations are as important as high VAF mutations, only a slight difference in the survival curves is expected; on the contrary, if the low VAF variants are not so critical for cancer progression, there might be significant differences in the survival^17^. In our cohort, no significant differences were observed between high-VAF-gene-predominant cases and low-VAF-gene-predominant cases in terms of survival. When the same criteria were applied to the MSKCC cohort, low-VAF-gene predominant cases showed worse prognosis (Fig. 7C). Even in our cohort, a significant association was found in some clinicopathologic parameters such as TNM stage (p = 0.037) and lymph node metastasis (p = 0.026), suggesting that high VAF genes might play a key role in these contextures.

Our study has some limitations. It has a relatively small sample size, a short follow-up time, and a retrospectively designed cohort. The frequency of NRAS mutation was too low to analyze the association between mutational status and clinical impact. In addition, the retrospectively designed cohort might have influenced the results. Thus, prospective randomized trials are warranted to validate these conclusions. Moreover, the gene alterations tested were only confined to those included in the commercial NGS panel, which might be overcame by the application of cancer panels in which greater numbers of potential genes are covered or WES in future studies. Besides, the results of germline mutation test for MMR genes are not available in this study.

## Materials and methods

### Tumor samples and DNA/RNA extraction

A total of 145 patients with CRC who previously underwent surgical resection in Seoul St. Mary’s hospital between 2016 and 2019 were enrolled. All cases were sporadic clinically, without any familial cancer syndromes. The clinicopathological parameters were retrospectively reviewed from the medical records. The study protocol was reviewed and approved by the institutional review board of The Catholic Medical Center, The Catholic University of Korea (IRB number: KC19RESI0669), and was performed in accordance with relevant guidelines and regulations. Informed consent was obtained from all participants and/or their legal guardians about this study.

CRC areas with rich tumor cell content (at least 70%) from unstained formalin-fixed, paraffin-embedded (FFPE) tissue specimens were obtained for microdissection. DNA and RNA were then extracted using the Recover All Total Nucleic Acid Isolation kit (Ambion, Thermo Fisher Scientific) according to the manufacturer’s instructions.

### Library preparation and sequencing

Library preparation was accomplished using the Ion Chef System and Oncomine Comprehensive Assay v1 and v3 (115 and 30 cases, respectively; Thermo Fisher Scientific, San Francisco, CA, USA) in accordance with the manufacturer’s instructions. Genomic DNA was then amplified, and targeted gene sequencing was performed using the Ion S5 XL sequencer (Thermo Fisher Scientific, San Francisco, CA, USA).

The sequencing data were analyzed with the Ion Torrent Suite version 5.10.2 (Life Technologies, San Francisco, CA, USA) using the variantCaller plugin. To eliminate base calling errors, several filtering steps were applied to generate the final variant calling: minimum allele frequency of hotspot variant: ≥ 4%, minimum allele frequency of indel variant: ≥ 5%, minimum allele frequency of SNP variant: ≥ 5%, minimum read counts for fusions: ≥ 40, CNV gain threshold: 4, and gain confidence level: 0.05. All identified variants were visually confirmed using the Integrative Genomics Viewer (IGV 2.8.6)^46^.

### MSI analysis

MSI assays were performed using paired normal and tumor samples as previously described^47^. The five microsatellite markers including two mononucleotide repeats (Bat-25 and Bat-26) and three dinucleotide repeats (D2S123, D5S346, and D17S250) recommended by the National Cancer Institute were amplified in a single multiplex PCR reaction. The PCR products were analyzed by capillary electrophoresis using an ABI 3500 automated sequencer (Applied Biosystems, Foster City, CA). Two of the five microsatellite markers demonstrated instability, and the tumor was considered to be high MSI (MSI-H). MSI at a single locus was defined as low MSI (MSI-L), while the absence of instability at any of the markers was defined as microsatellite stable (MSS). For statistical purposes, MSI-L tumors were considered together with MSS tumors, because of the similarity between MSI-L and MSS tumors^48^.

### Public datasets

In this study, we evaluated the clinicopathologic and genomic data from two public datasets and compared these data with those of our cohort.

The TCGA program offers great opportunity to identify the genotype–phenotype relationship, providing extensive archives of multi-omics data. We assessed the somatic mutation data of 459 patients with CRC (colon cancer: 341 and rectal cancer: 118) from the TCGA cohort. The TCGA data were downloaded from the Genomic Data Commons Data Portal (GDC portal, https://portal.gdc.cancer.gov/). In addition, data matrices and supporting data from two flagship articles (version 20120719 and version 20180409) were also downloaded^10,49^.

The MSKCC cohort contains a large-scale, prospective clinical sequencing data established by conducting MSK-IMPACT, a hybridization capture-based NGS assay for targeted deep sequencing of 341 key cancer genes, in 10,336 patients with 62 principal cancer types^11^. We downloaded the supplementary information and source data files available in the online version of the article, which include the data of 1,003 patients with CRCs.

### Statistical analysis

All statistical analyses were performed using a combination of R 4.0.2 for Windows (R Core Team 2020, A language and environment for statistical computing, R Foundation for Statistical Computing, Vienna, Austria, URL http://www.r-project.org/) and online chi-square/Fisher’s exact test software (GraphPad, www.graphpad.com/quickcalcs/contingency1/). Lollipop plots and somatic mutation plots were depicted using MAF tools^50^. The chi-square test with Yates' correction or the chi-square test for association was used to determine the relationship between the clinicopathological characteristics and gene mutations. OS was defined as the period between initial diagnosis and death from any cause or last follow-up visit. Disease-free survival (DFS) was defined as the period between initial diagnosis and detection of recurrence or metastasis. Survival was analyzed by the Kaplan*–*Meier method using the log-rank test. Multivariate survival analyses and calculation of hazard ratios (HRs) were performed using the Cox proportional hazards model to determine the independent predictors of OS. A two-sided p-value of < 0.05 was considered significant.

## Conclusions

In conclusion, this study presented a mutational landscape of actionable genes in CRC, validating the prognostic role of genomic alterations in TP53, BRAF, ATM, KRAS, and FBXW7 as well as MSI status. In addition, we investigated the clinical relevance of low VAF variants. A comprehensive analysis of molecular markers for CRC can provide insights into the disease progression and help optimize a personalized therapy in the Korean population.

## Supplementary Information

Supplementary Information

## Abbreviations

CRC: Colorectal cancer
OS: Overall survival
DFS: Disease-free survival
EGFR: Epidermal growth factor receptor
MSI: Microsatellite instability
NGS: Next-generation sequencing
VAF: Variant allele frequency
TCGA: The Cancer Genome Atlas
MSKCC: Memorial Sloan Kettering Cancer Center
