# Supplementary material for: Enhancing the landscape of colorectal cancer using targeted deep sequencing
Source: Sci Rep. 2021 Apr 14;11:8154. doi: 10.1038/s41598-021-87486-3 (PMC8046812; doi:10.1038/s41598-021-87486-3)
Supplement: Supplementary file 1 — Supplementary Information [file 41598_2021_87486_MOESM1_ESM.docx]

SUPPLEMENTARY MATERIAL

Enhancing the landscape of colorectal cancer using targeted deep sequencing

Chul Seung Lee ^1^, In Hye Song ^2^, Ahwon Lee ^2^, Jun Kang ^2^, Yoon Suk Lee ^1^, In Kyu Lee ^1^, Young Soo Song ^3,^*, Sung Hak Lee ^2,^*

Appendix A

Figure A1. Oncoplot of the top 15 most frequently mutated genes in the TCGA cohort. The plot provided an overview of somatic mutations in particular genes (rows) affecting individual samples (columns). It shows 223 positive samples. TCGA, The Cancer Genome Atlas

Figure A2. Oncoplot of the top 20 most frequently mutated genes in the MSKCC cohort. There are 970 positive samples. MSKCC, Memorial Sloan Kettering Cancer Center

Figure A3. The variant prevalence and spectrum of TP53 (A), KRAS (B), APC (C), PIK3CA (D), SMAD4 (E) genes in TCGA cohort. All graphs depict a lollipop plot showing identified variants relative to a schematic representation of the gene. Y-axis represent total number of mutations at each residue.

Figure A4. The variant prevalence and spectrum of TP53 (A), KRAS (B), APC (C), PIK3CA (D), SMAD4 (E) genes in MSKCC cohort. All graphs depict a lollipop plot showing identified variants relative to a schematic representation of the gene. Y-axis represent total number of mutations at each residue.

Figure A5. Mutually exclusive and co-occurring gene pairs in the TCGA dataset presented in a triangular matrix. Mutually exclusive/co-occurrence event on top 25 mutated genes are shown. Bluish green indicates tendency toward co-occurrence, whereas brown indicates tendency toward mutually exclusiveness.

Figure A6. Mutually exclusive and co-occurring gene pairs in MSKCC dataset presented in a a triangular matrix. The mutually exclusive/co-occurrence event in the 25 mutated genes are shown. Bluish green indicates a tendency toward co-occurrence, whereas brown indicates a tendency toward mutually exclusiveness.

Figure A7. Kaplan–Meier curve for OS in stage 3 and 4 population by mutational status, including ATM (A) and BRAF (B).

Figure A8. Kaplan–Meier curve for DFS in stage 3 and 4 population by mutational status, including FBXW7 (A) and TP53 (B)

Figure A9. Kaplan–Meier curve for OS in the TCGA dataset by mutational status, including TP53 (A), KRAS (B), APC (C), PIK3CA (D), SMAD4 (E), BRAF (F), NRAS (G), ATM (H), and FBXW7 (I)

Figure A10. Kaplan–Meier curve for OS in the MSKCC dataset by mutational status, including TP53 (A), KRAS (B), APC (C), PIK3CA (D), SMAD4 (E), BRAF (F), NRAS (G), ATM (H), and FBXW7 (I)

Figure A11. Distribution of VAFs (*Y*-axis) of SNPs (*X*-axis) among the top mutated genes in our study dataset. The numbers on upper part of the figure correspond to mutated patients.

Figure A12. Comparison of the distribution of VAF for validated somatic mutations between the TCGA cohort and our cohort. The density is estimated by Gaussian kernel.

Figure A13. Scatterplot representing the relationships between median VAF of genes and occurrence of corresponding genes (A) and boxplot of VAF according to mutation types in the MSKCC cohort (B). Genes with less than three instances of occurrences are excluded. The red line indicates the results of linear regression. SNP: Single nucleotide polymorphism; DNP: Double nucleotide polymorphism; TNP: Triple nucleotide polymorphism; ONP: Oligo-nucleotide polymorphism; INS: Insertion; DEL: Deletion

Figure A14. Kaplan–Meier curve for OS (A) and DFS (B) in our cohort by stages among MSI-L/MSS tumors

Figure A15. Kaplan–Meier curve for OS (A) and DFS (B) in our cohort by M stage among MSI-L/MSS tumors

Figure A16. Kaplan–Meier curve for OS in the TCGA dataset by overall stages (A) and M stage (B)

Appendix B

Table A1. Mutation spectrum of driver genes detected in our cohort.

Table A2. Mutation spectrum of driver genes in the TCGA cohort

Table A3. Mutation spectrum of driver genes in the MSKCC cohort

Table A4. Correlation of common gene mutations with tumor stages in TCGA cohort

Table A5. Classification of genes according to median VAF

Table A6. Correlation of MSI status according to the clinicopathological data and KRAS, NRAS, PIK3CA, and BRAF mutations in the TCGA cohort

Table A7. The MMR protein and BRAF and MMR gene mutation status in MSI-H group

Appendix A


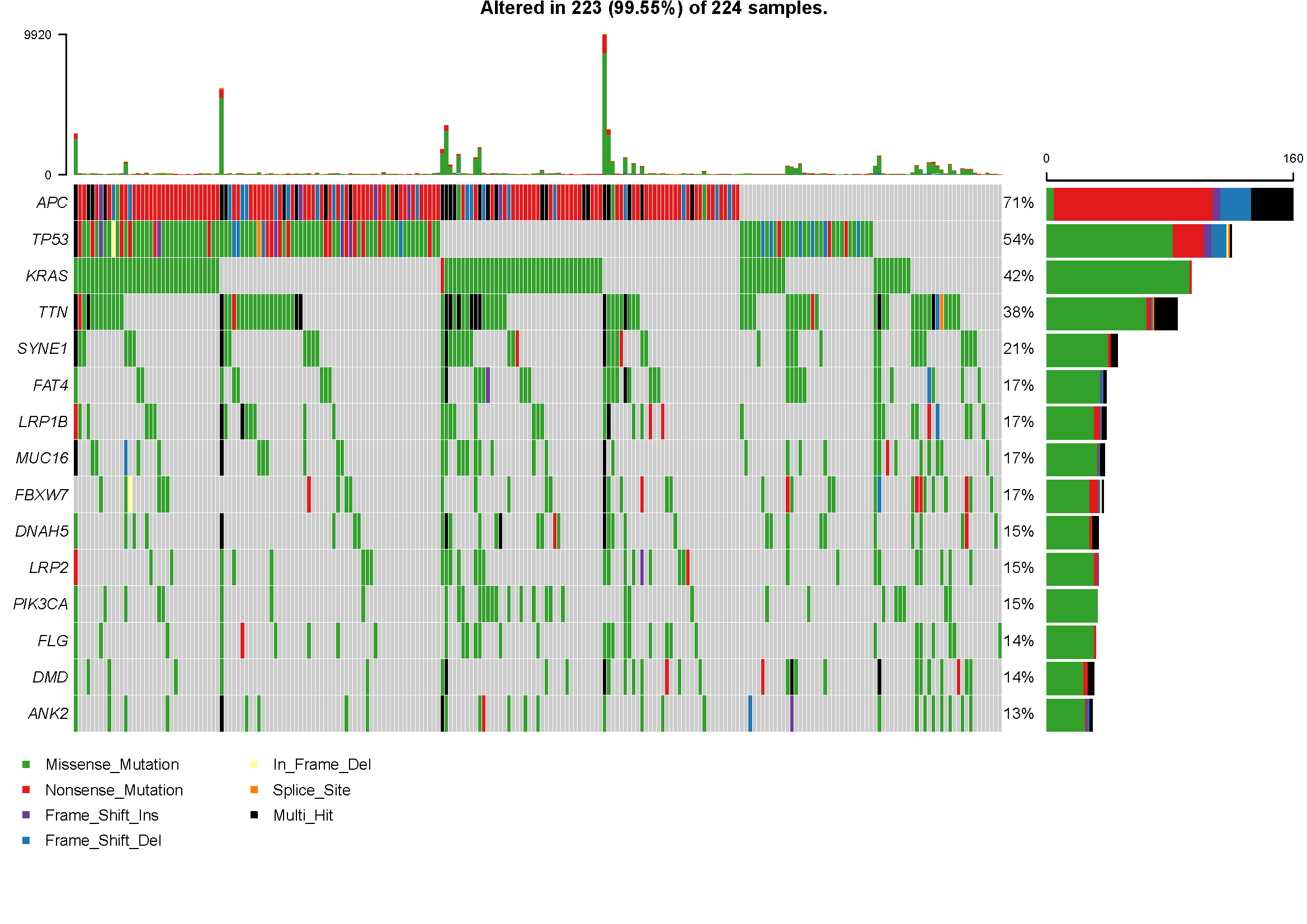


Figure A1. Oncoplot of the top 15 most frequently mutated genes in the TCGA cohort. The plot provided an overview of somatic mutations in particular genes (rows) affecting individual samples (columns). It shows 223 positive samples. TCGA, The Cancer Genome Atlas


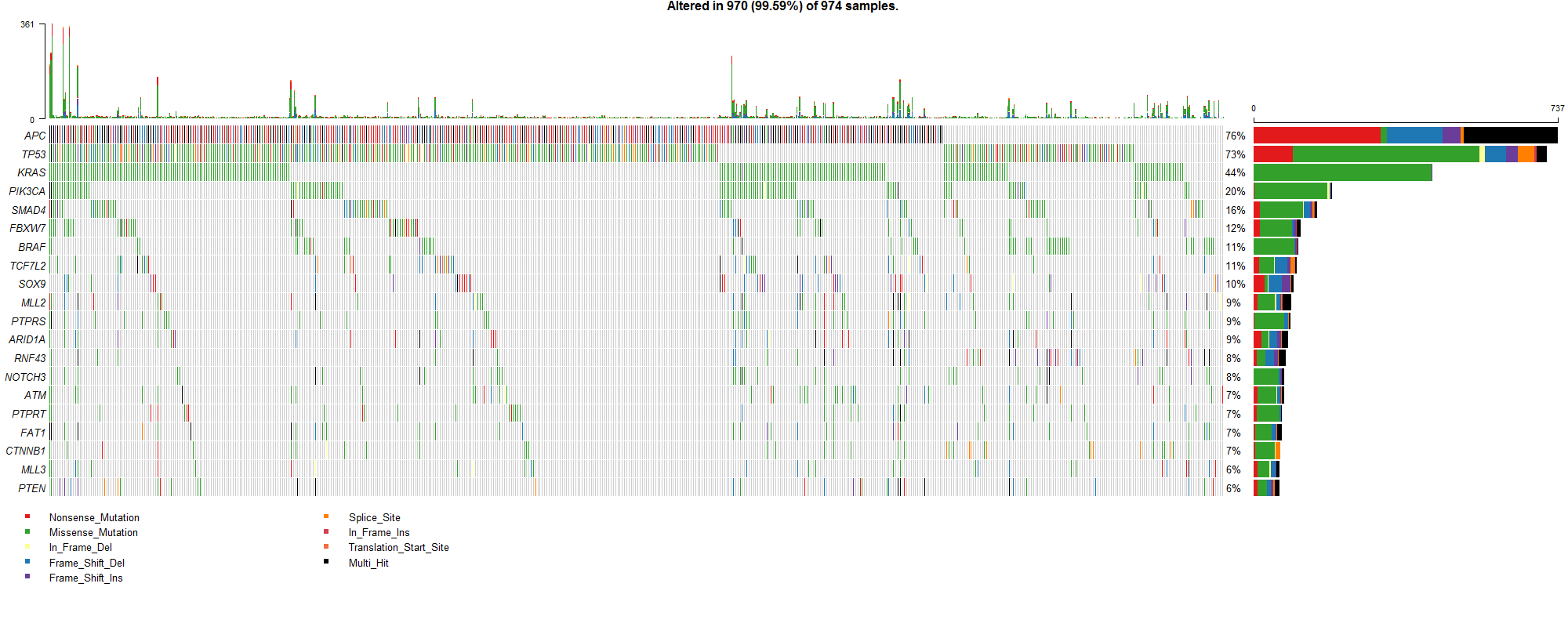


Figure A2. Oncoplot of the top 20 most frequently mutated genes in the MSKCC cohort. There are 970 positive samples. MSKCC, Memorial Sloan Kettering Cancer Center

| 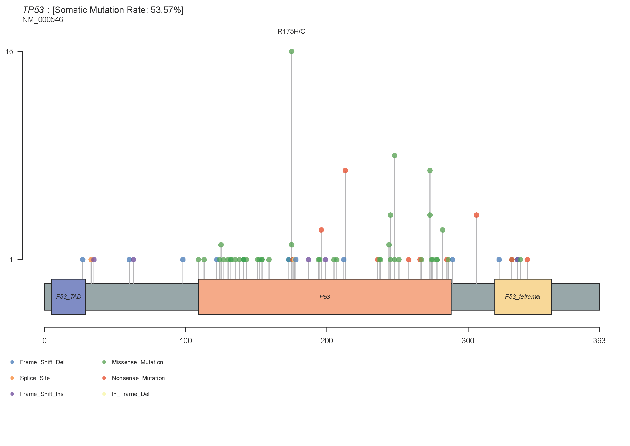A | 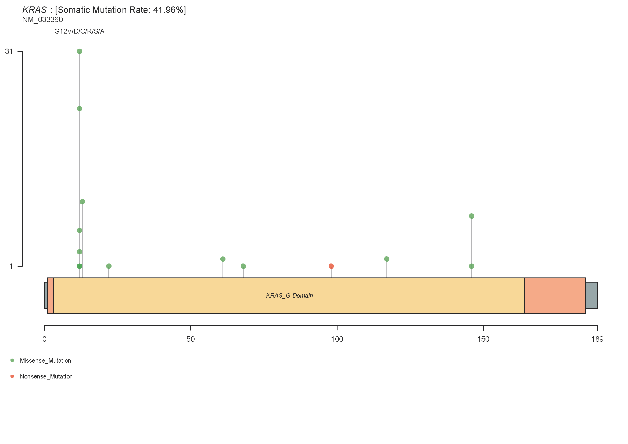B |
| --- | --- |
| 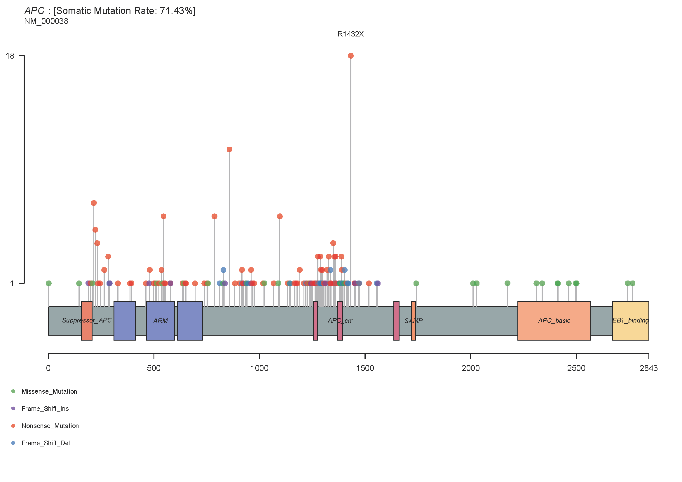C | 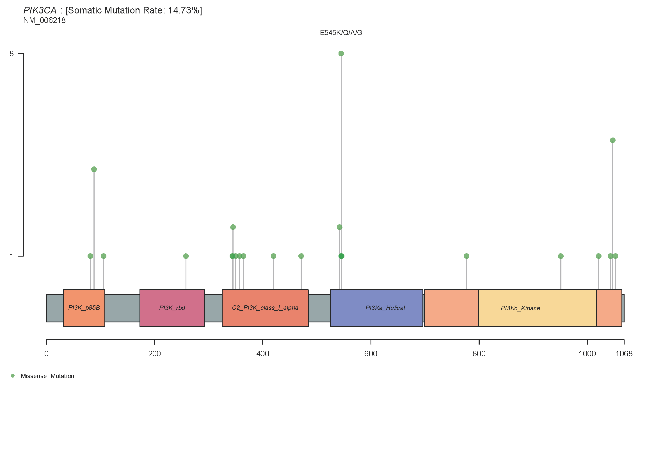D |
| 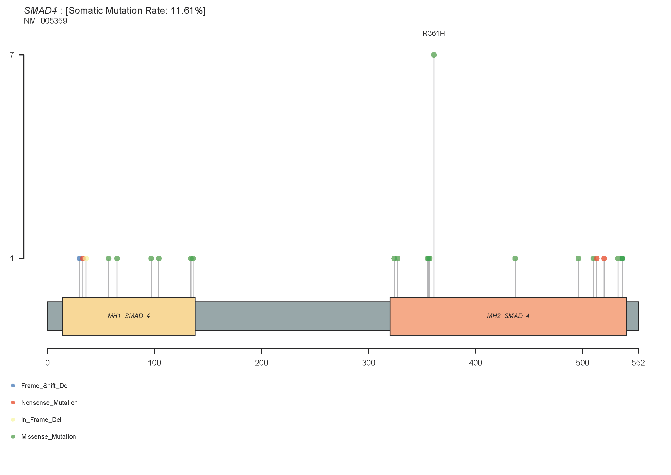E |  |

Figure A3. The variant prevalence and spectrum of TP53 (A), KRAS (B), APC (C), PIK3CA (D), SMAD4 (E) genes in TCGA cohort. All graphs depict a lollipop plot showing identified variants relative to a schematic representation of the gene. Y-axis represent total number of mutations at each residue.

| 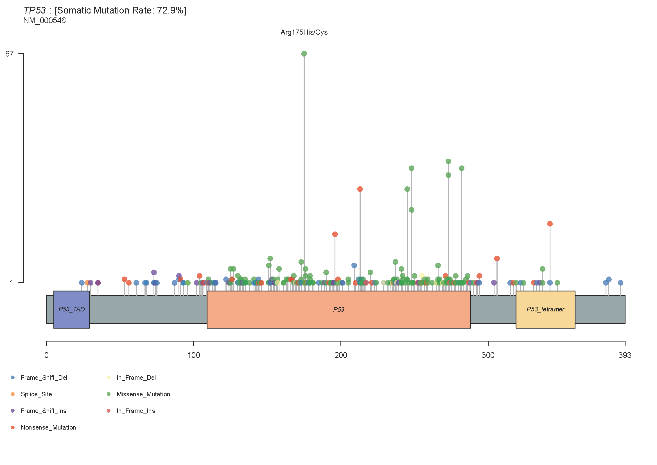A | 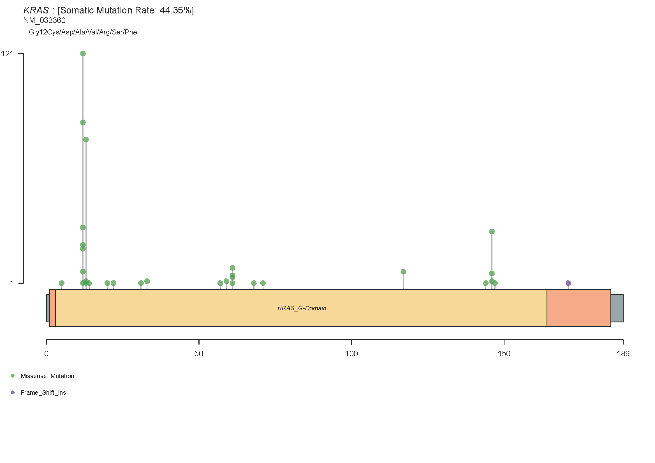B |
| --- | --- |
| 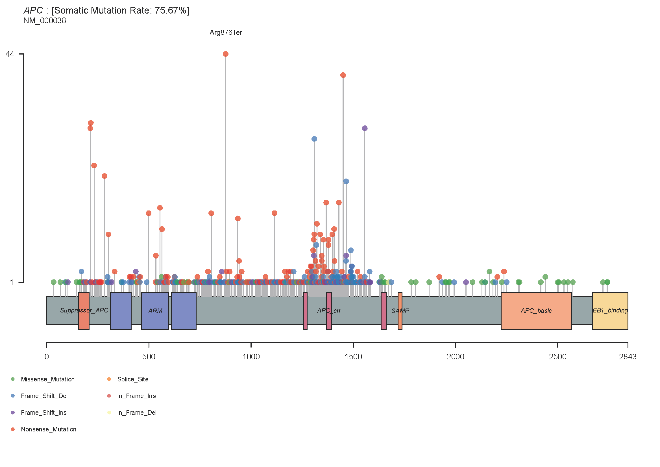C | 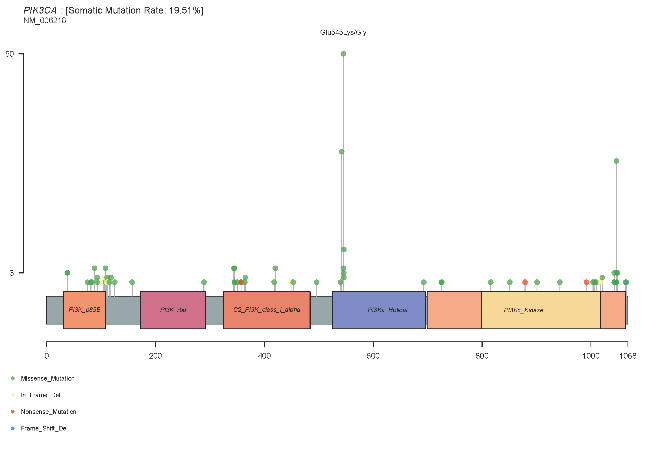D |
| 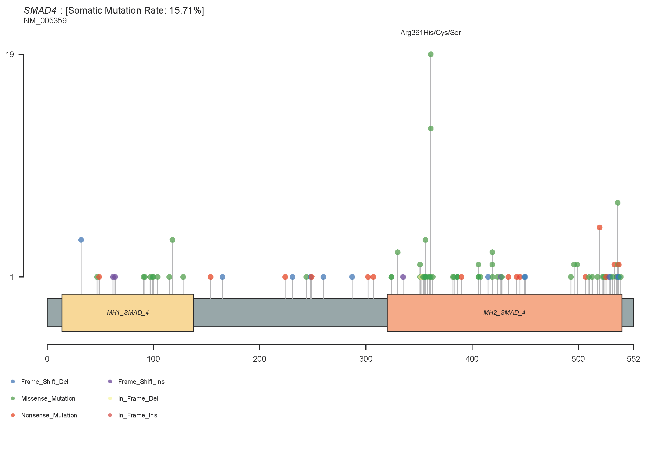E |  |

Figure A4. The variant prevalence and spectrum of TP53 (A), KRAS (B), APC (C), PIK3CA (D), SMAD4 (E) genes in MSKCC cohort. All graphs depict a lollipop plot showing identified variants relative to a schematic representation of the gene. Y-axis represent total number of mutations at each residue.


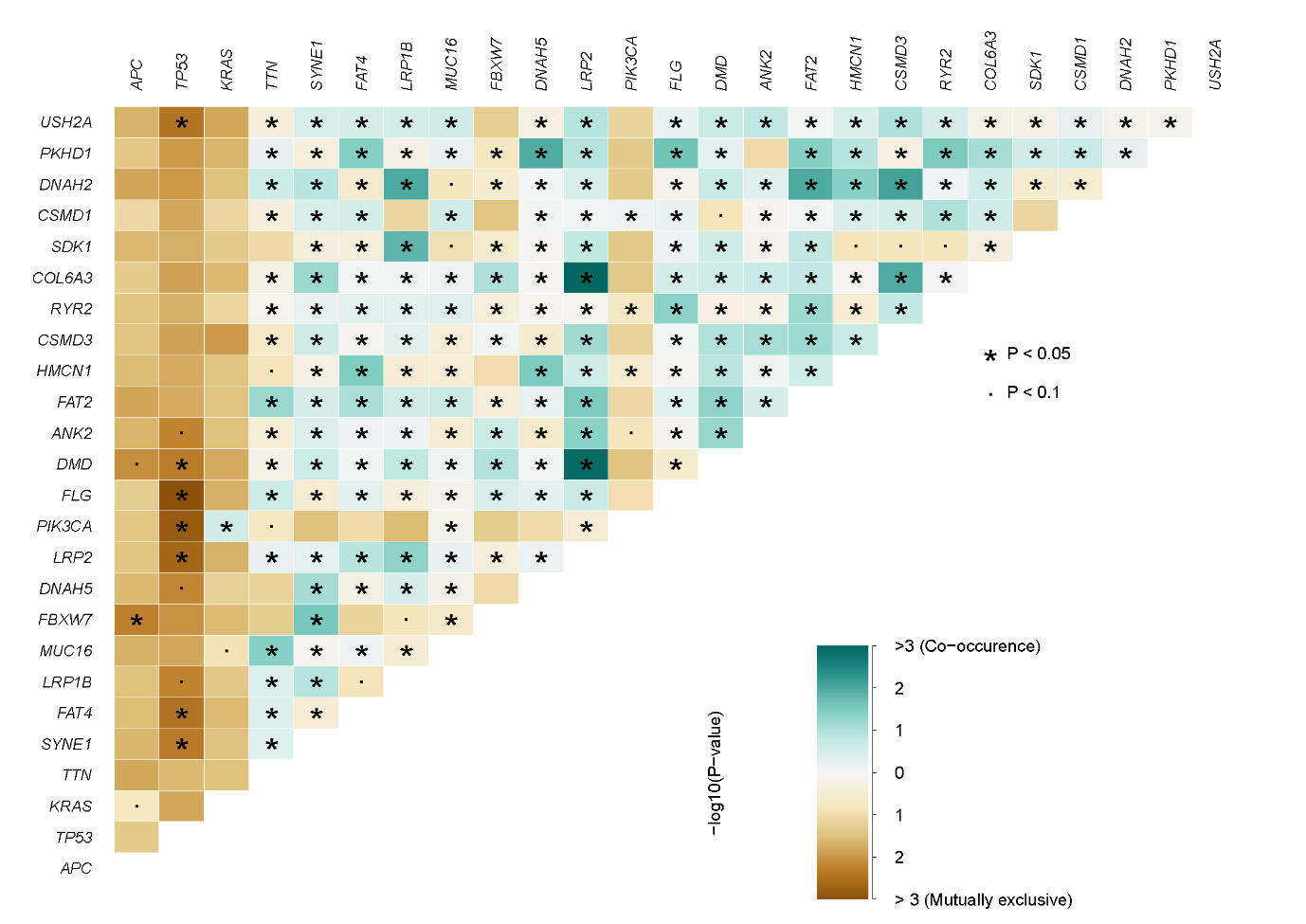


Figure A5. Mutually exclusive and co-occurring gene pairs in the TCGA dataset presented in a triangular matrix. Mutually exclusive/co-occurrence event on top 25 mutated genes are shown. Bluish green indicates tendency toward co-occurrence, whereas brown indicates tendency toward mutually exclusiveness.


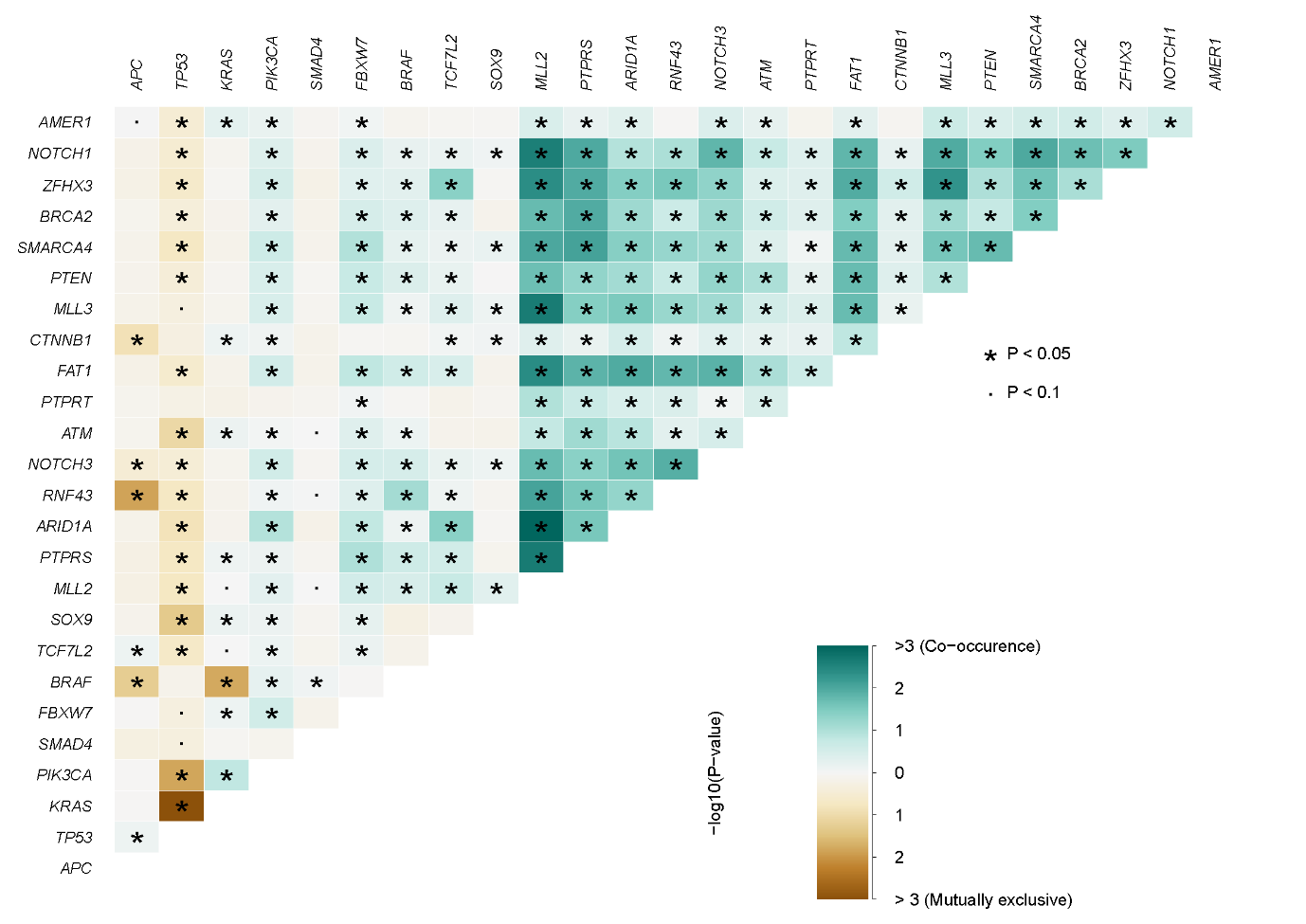


Figure A6. Mutually exclusive and co-occurring gene pairs in MSKCC dataset presented in a a triangular matrix. The mutually exclusive/co-occurrence event in the 25 mutated genes are shown. Bluish green indicates a tendency toward co-occurrence, whereas brown indicates a tendency toward mutually exclusiveness.

| 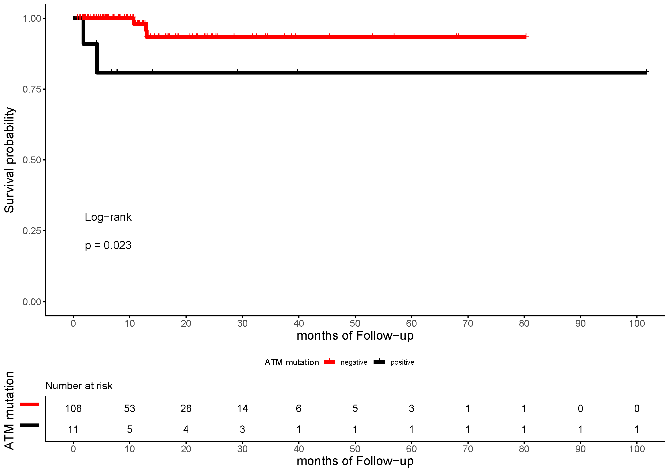A | 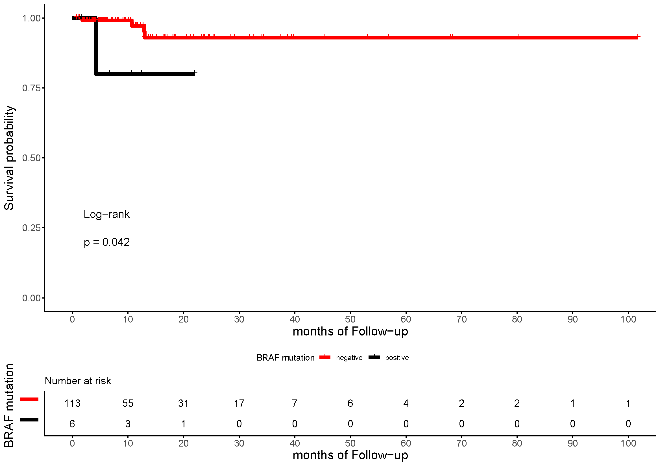B |
| --- | --- |

Figure A7. Kaplan–Meier curve for OS in stage 3 and 4 population by mutational status, including ATM (A) and BRAF (B).

| 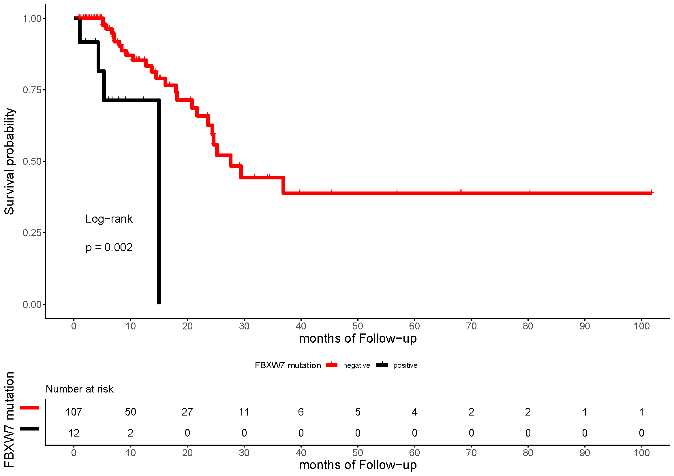A | 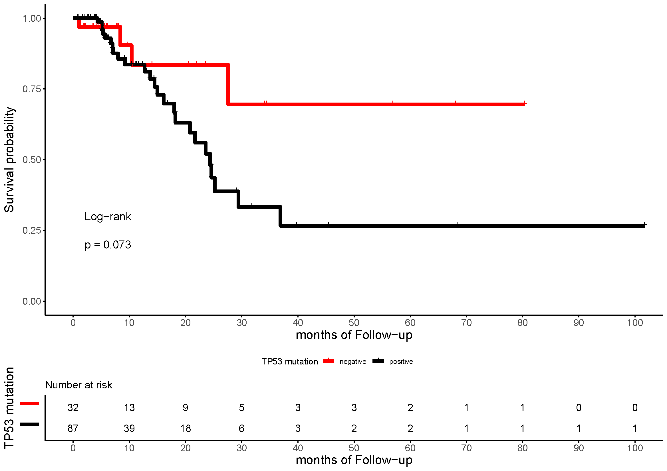B |
| --- | --- |

Figure A8. Kaplan–Meier curve for DFS in stage 3 and 4 population by mutational status, including FBXW7 (A) and TP53 (B)

| 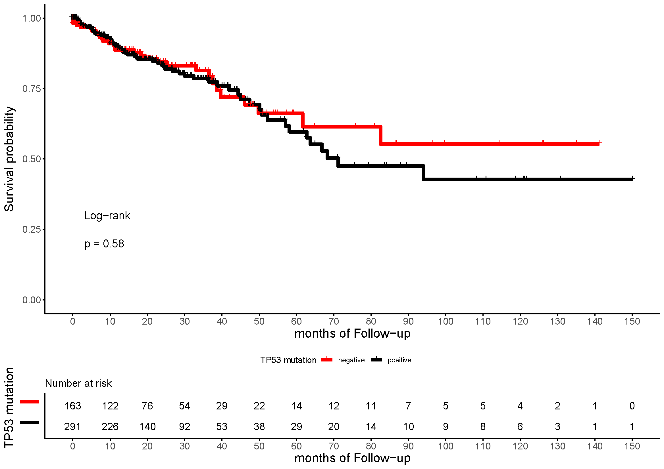A | 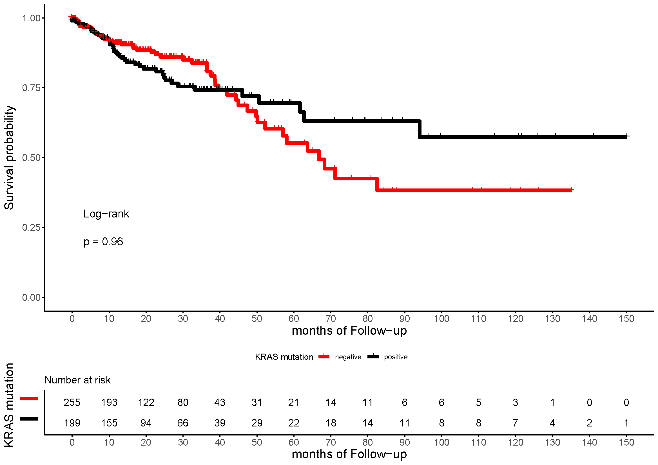B |
| --- | --- |
| 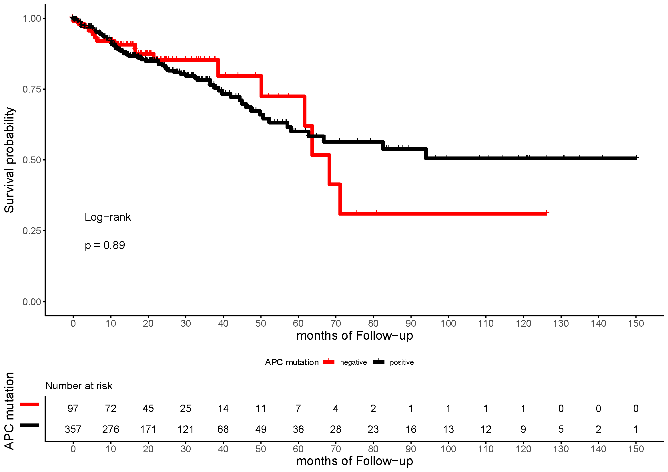C | 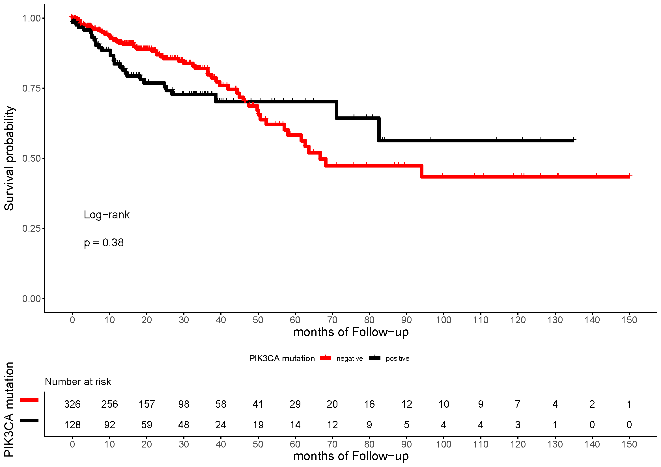D |
| 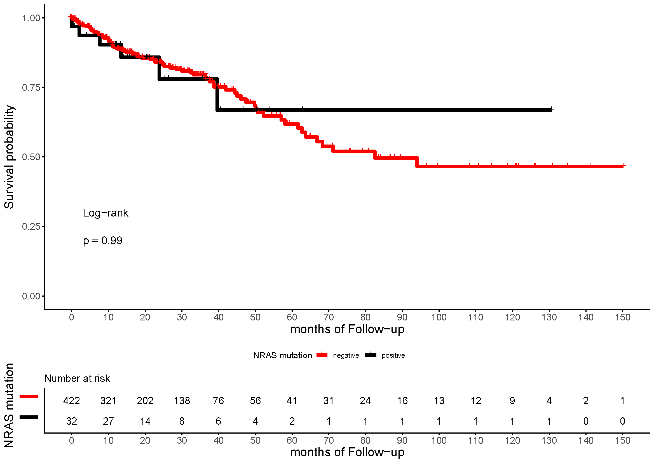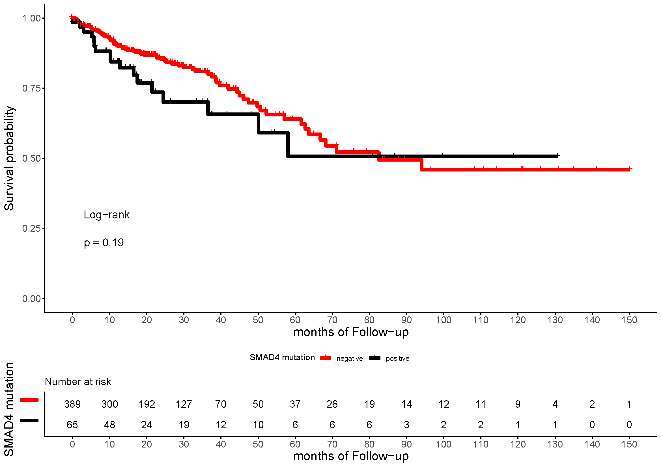E | 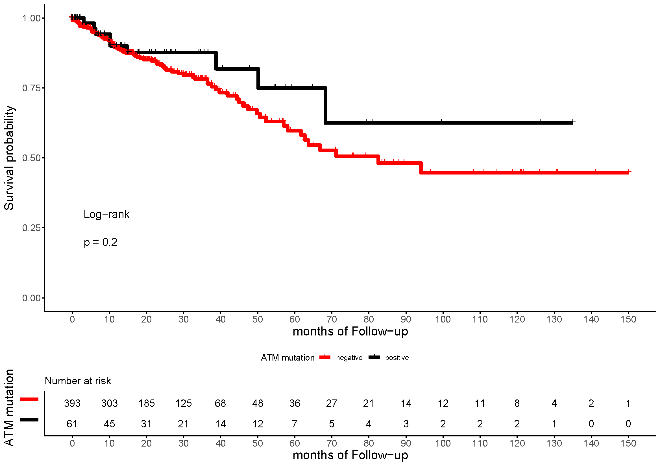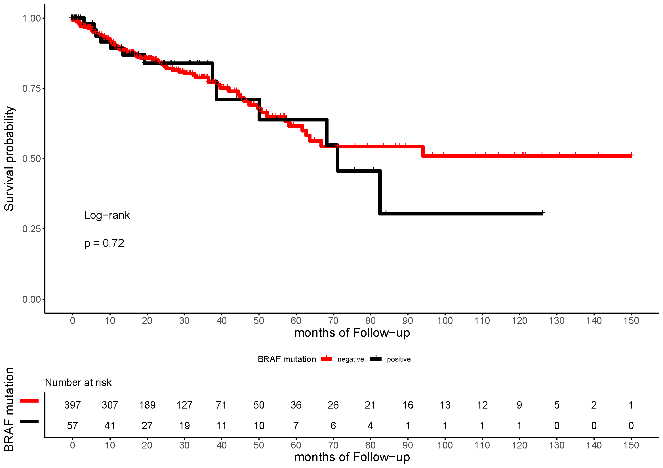F |
| G | H |
| 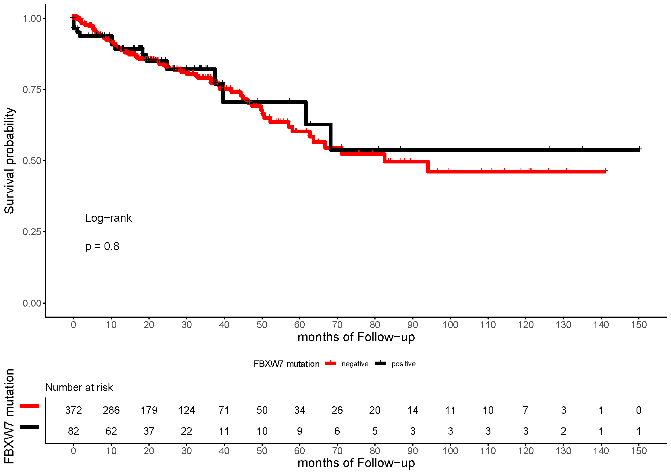I |  |

Figure A9. Kaplan–Meier curve for OS in the TCGA dataset by mutational status, including TP53 (A), KRAS (B), APC (C), PIK3CA (D), SMAD4 (E), BRAF (F), NRAS (G), ATM (H), and FBXW7 (I)

| 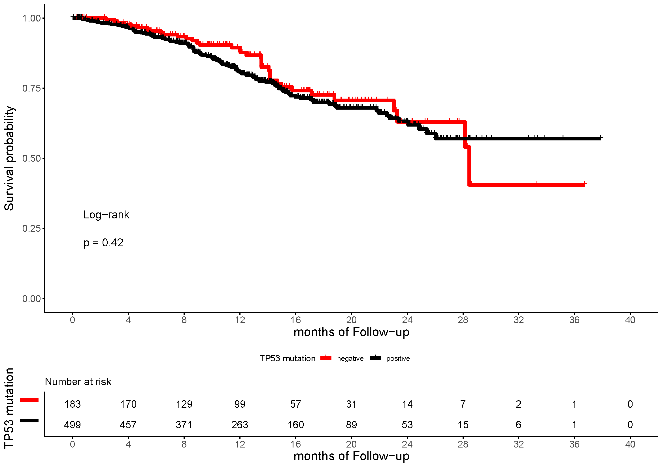A | 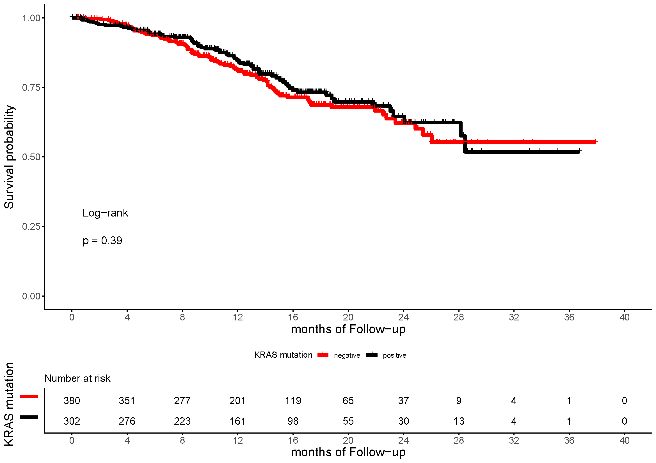B |
| --- | --- |
| 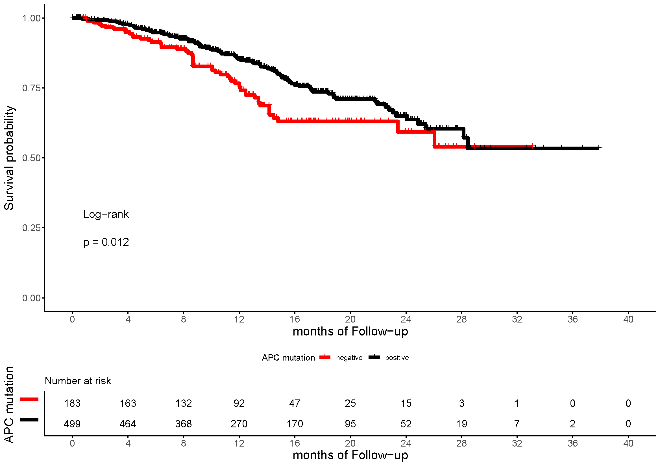C | 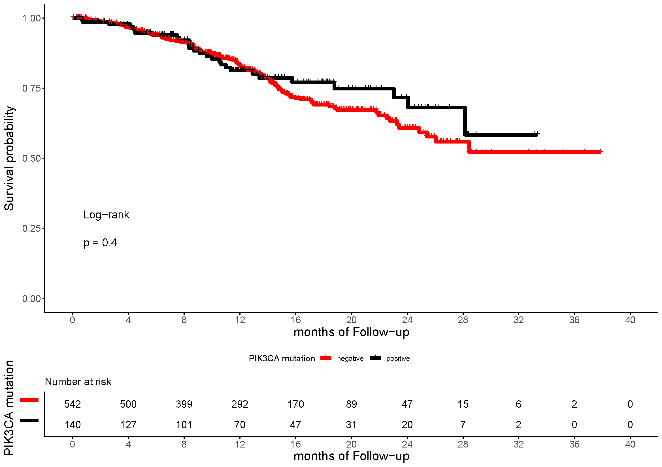D |
| 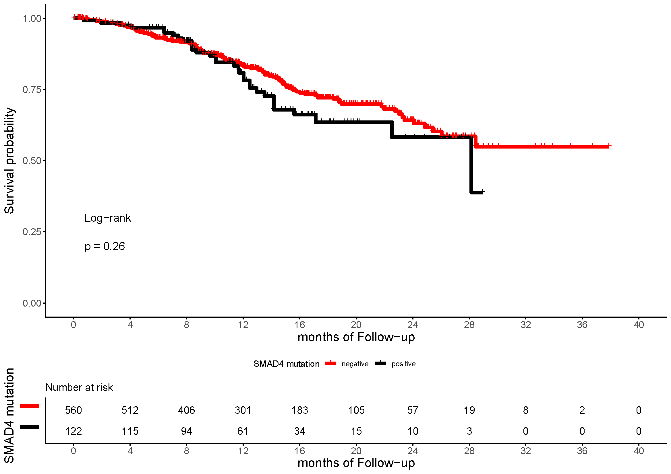E | 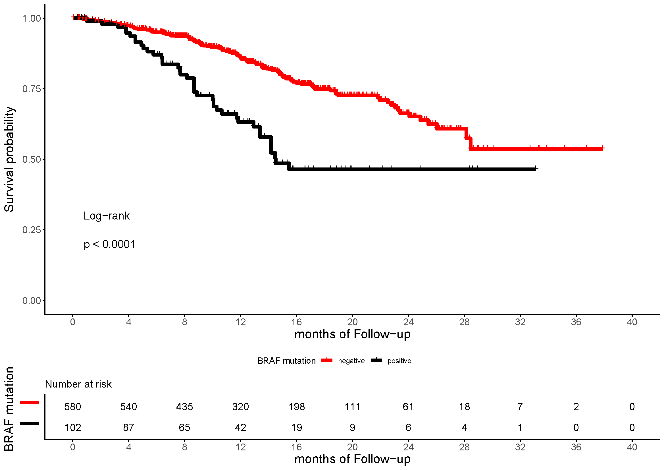F |
| 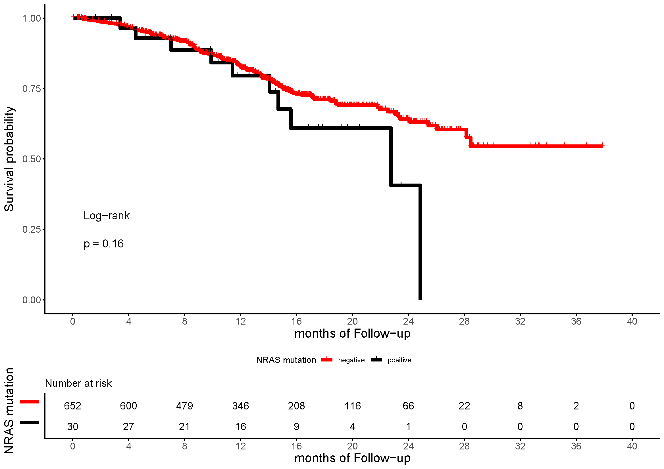G | 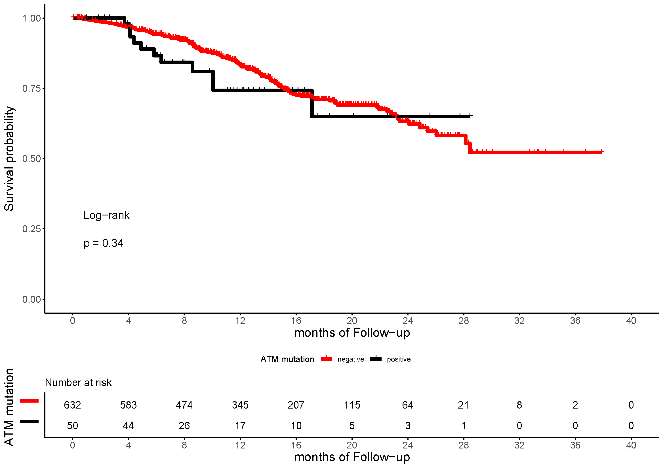H |
| 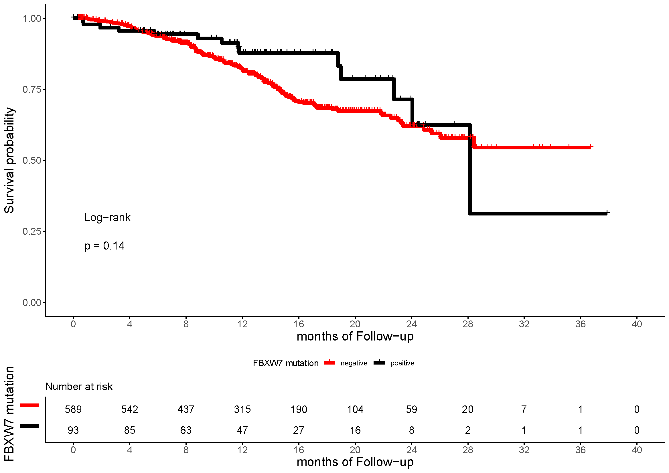I |  |

Figure A10. Kaplan–Meier curve for OS in the MSKCC dataset by mutational status, including TP53 (A), KRAS (B), APC (C), PIK3CA (D), SMAD4 (E), BRAF (F), NRAS (G), ATM (H), and FBXW7 (I)


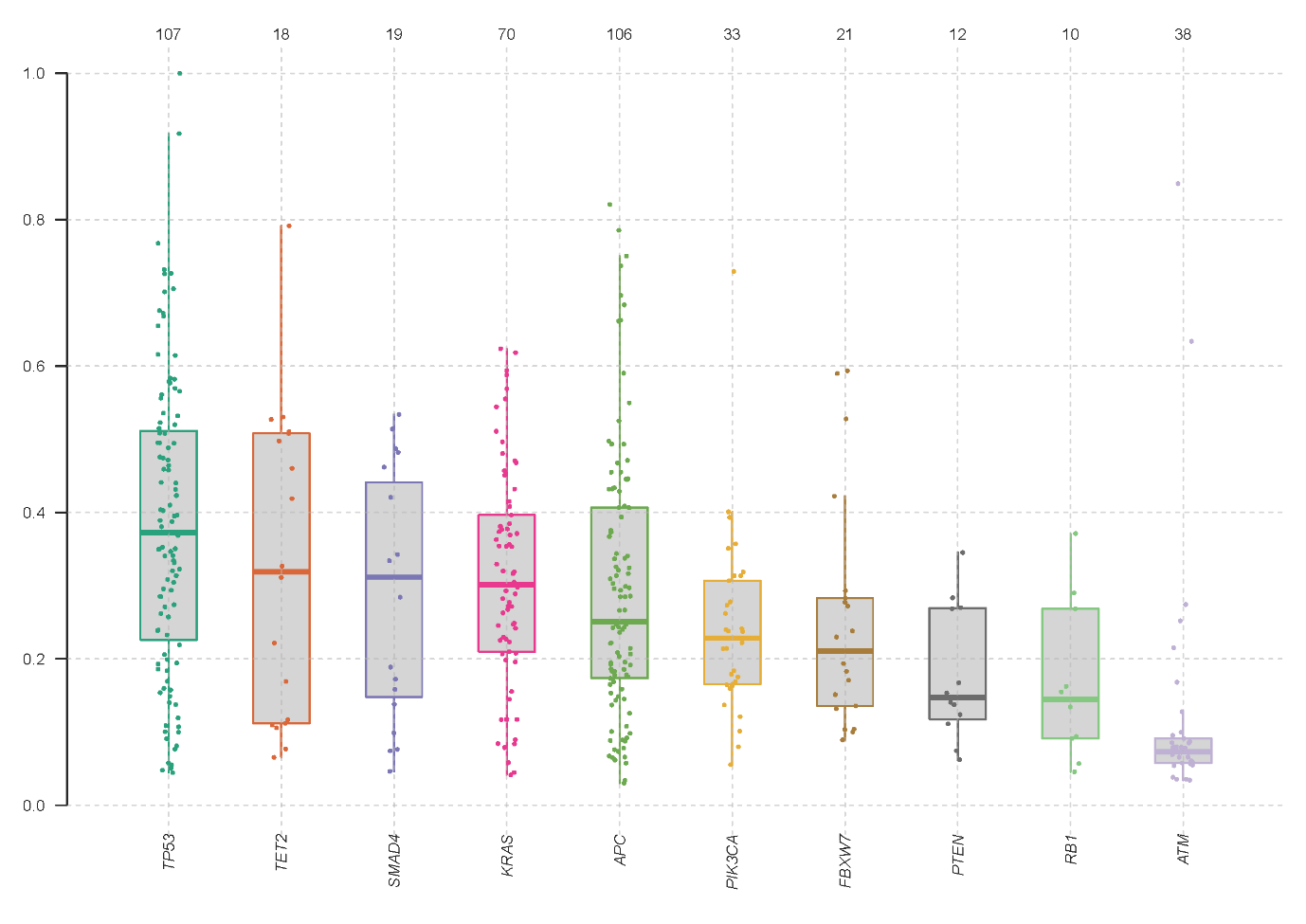


Figure A11. Distribution of VAFs (*Y*-axis) of SNPs (*X*-axis) among the top mutated genes in our study dataset. The numbers on upper part of the figure correspond to mutated patients.


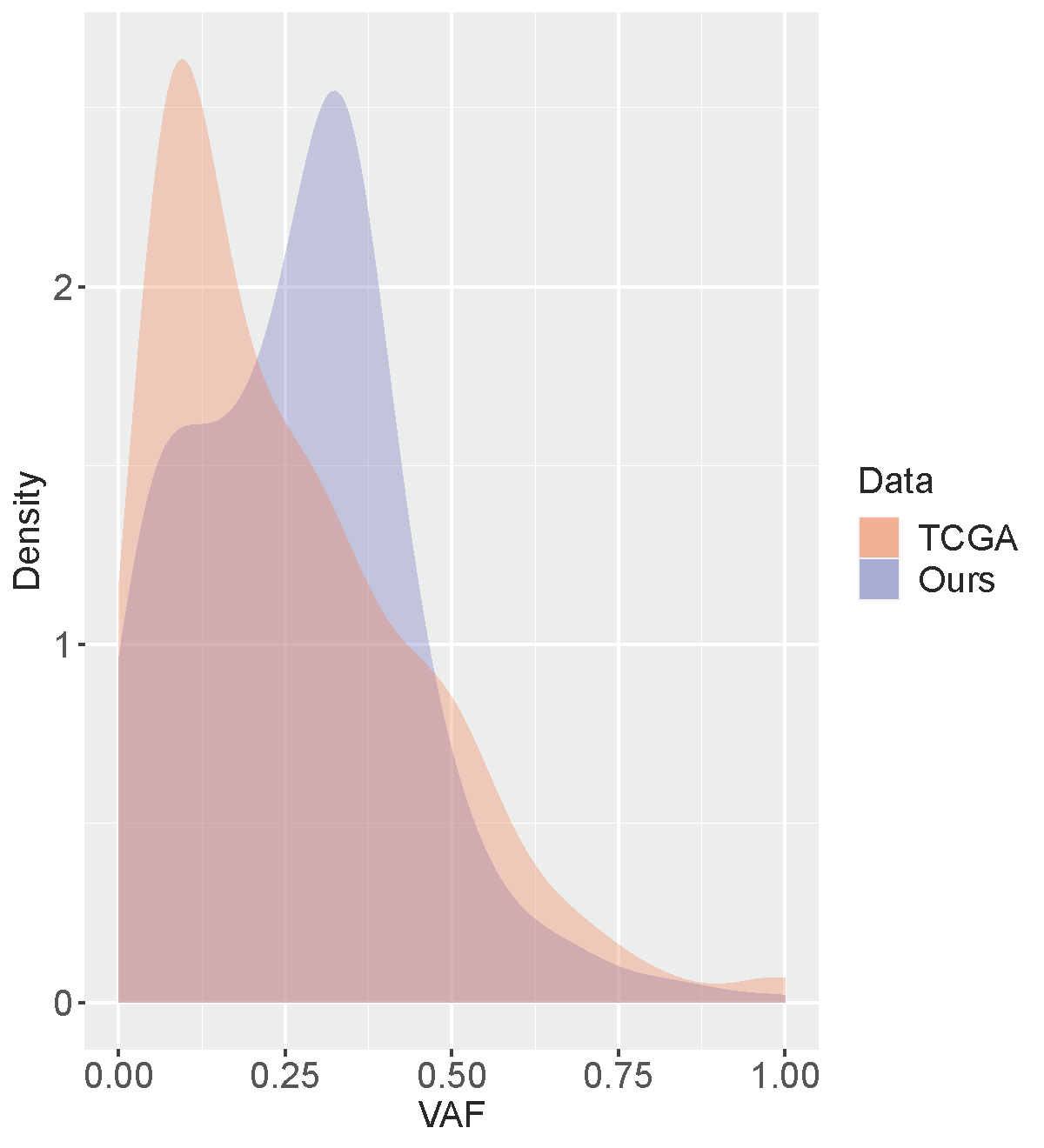


Figure A12. Comparison of the distribution of VAF for validated somatic mutations between the TCGA cohort and our cohort. The density is estimated by Gaussian kernel.

| 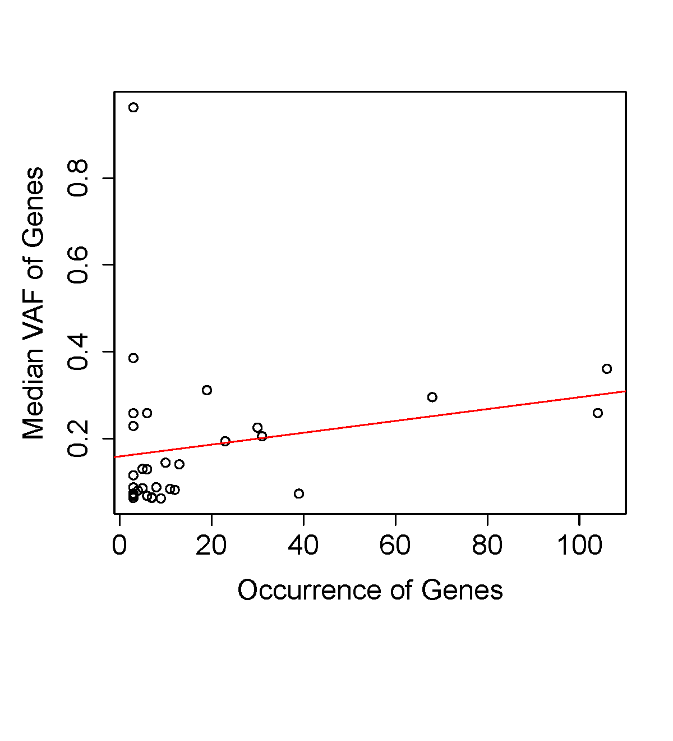A | B |
| --- | --- |

Figure A13. Scatterplot representing the relationships between median VAF of genes and occurrence of corresponding genes (A) and boxplot of VAF according to mutation types in the MSKCC cohort (B). Genes with less than three instances of occurrences are excluded. The red line indicates the results of linear regression. SNP: Single nucleotide polymorphism; DNP: Double nucleotide polymorphism; TNP: Triple nucleotide polymorphism; ONP: Oligo-nucleotide polymorphism; INS: Insertion; DEL: Deletion

| 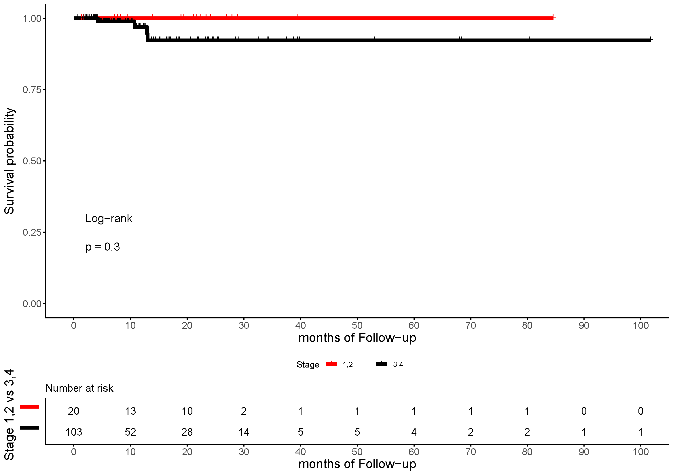A | 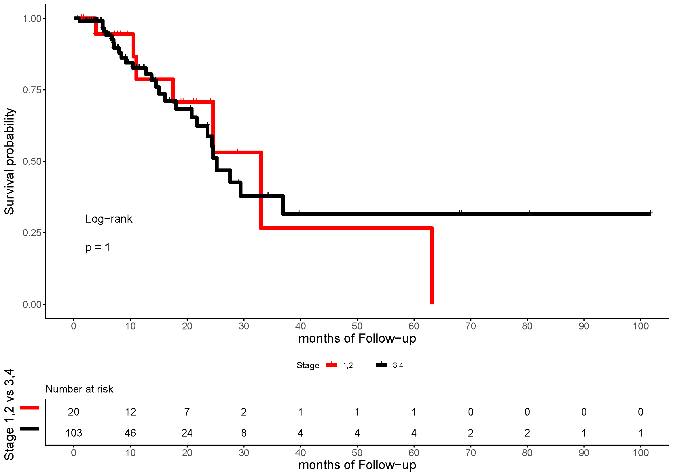B |
| --- | --- |

Figure A14. Kaplan–Meier curve for OS (A) and DFS (B) in our cohort by overall stages among MSI-L/MSS tumors

| 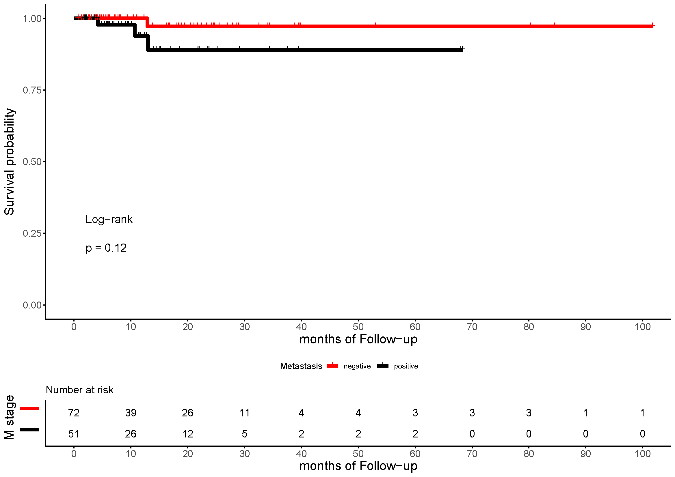A | 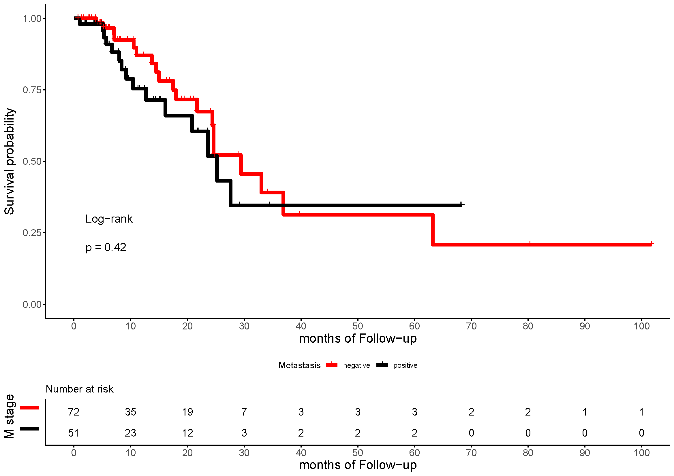B |
| --- | --- |

Figure A15. Kaplan–Meier curve for OS (A) and DFS (B) in our cohort by M stage among MSI-L/MSS tumors

| 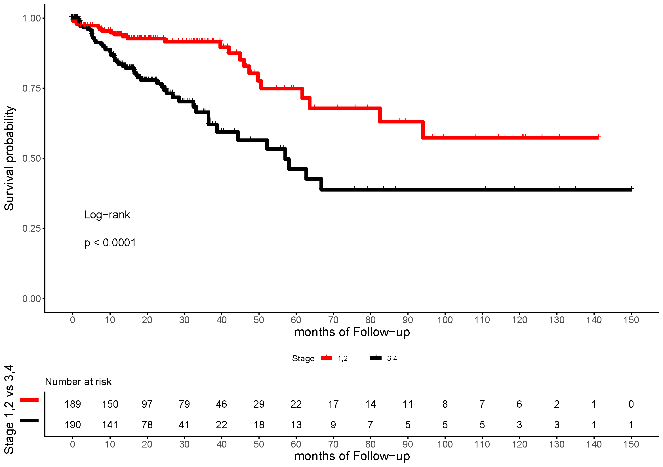A | 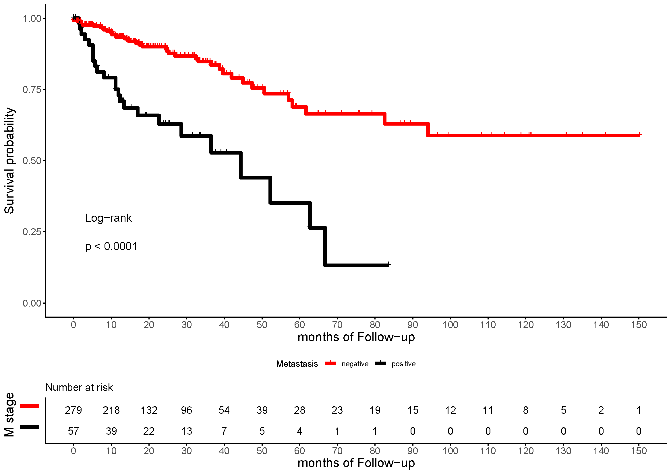B |
| --- | --- |

Figure A16. Kaplan–Meier curve for OS in the TCGA dataset by overall stages (A) and M stage (B)

Appendix B

**Table A1.** Mutation spectrum of driver genes detected in our cohort.

| **Gene** | **Cases (Variants)** | **Clinical significance** | **Variation** |
| --- | --- | --- | --- |
| TP53 | 100 (107) | Pathogenic/ Likely pathogenic (n) | p.Arg175His (8), p.Arg196Ter (2), p.Arg209fs (1), p.Arg213Ter (1), p.Arg248Gln (4), p.Arg248Trp (7), p.Arg273Cys (9), p.Arg273His (4), p.Arg280fs (1), p.Arg282Gly (1), p.Arg282Trp (3), p.Arg306Ter (3), p.Arg337fs (1), p.Arg342Ter (2), p.Asn131fs (1), p.Cys124fs (1), p.Cys176Phe (1), p.Cys238Tyr (2), p.Cys275Phe (1), p.Gln167fs (1), p.Gln52fs (1), p.Glu285fs (1), p.Glu285Lys (4), p.Glu339Ter (1), p.Gly244Asp (1), p.Gly244Ser (2), p.Gly245Asp (2), p.Gly245Ser (3), p.Gly266fs (1), p.Ile195Thr (1), p.Leu257Gln (1), p.Met133fs (1), p.Ser241Tyr (1), p.Trp53Ter (1), p.Trp91Ter (1), p.Tyr220Asn (1), p.Tyr220Cys (2), p.Tyr234Cys (1), p.Val122fs (1), p.Val173Leu (1), p.Val73fs (1) |
|  |  | Uncertain significance (n) | p.Arg249Ser (1), p.Gly244Cys (1), p.Gly262Val (1), p.Gly266Arg (1), p.Gly266Glu (1), p.Lys132Arg (1), p.Lys132Asn (1), p.Met237Ile (1), p.Pro250Leu (1), p.Tyr163His (1) |
|  |  | Not provided (n) | p.Asn239_Ser240del (1), p.Cys135Ter (1), p.Glu224Ter (1), p.Glu258Asp (1), p.Glu287Ter (1), p.Glu326fs (1), p.Lys292Ter (1), p.Lys320fs (1), p.Ser240Cys (1), p.Ser240Ile (1), p.Thr256Lys (1), p.Tyr220fs (1), p.Tyr220Ter (1), p.Tyr236fs (1) |
| KRAS | 75 (75)^a^ | Pathogenic/ Likely pathogenic (n) | p.Ala146Val (1), p.Ala59Thr (2), p.Gln61His (1), p.Gln61Leu (1), p.Gln61Lys (2), p.Gly12Ala (3), p.Gly12Asp (29), p.Gly12Cys (3), p.Gly12Ser (3), p.Gly12Val (7), p.Gly13Asp (14) |
|  |  | Uncertain significance (n) | p.Ala146Thr (3) |
|  |  | Not provided (n) | p.Lys117Leu (1) |
| APC | 67 (106) | Pathogenic/ Likely pathogenic (n) | p.Arg1114Ter (3), p.Arg1450Ter (5), p.Arg213Ter (6), p.Arg216Ter (1), p.Arg2204Ter (2), p.Arg2237Ter (1), p.Arg232Ter (3), p.Arg283Ter (2), p.Arg405Ter (1), p.Arg499Ter (2), p.Arg564Ter (4), p.Arg876Ter (2), p.Asn1455fs (1), p.Asn1830fs (1), p.Asn659fs (1), p.Gln1338Ter (2), p.Gln1367Ter (1), p.Gln1378Ter (2), p.Gln1429Ter (5), p.Gln473Ter (2), p.Glu1306Ter (1), p.Glu1309fs (2), p.Glu1309Ter (1), p.Glu1353Ter (1), p.Leu1488fs (1), p.Lys1030Ter (1), p.Lys1199fs (1), p.Ser1044Ter (1), p.Ser105fs (1), p.Ser1298fs (1), p.Ser1465fs (2), p.Ser1857fs (1), p.Ser457Ter (3), p.Tyr1075Ter (1), p.Tyr935Ter (2), p.Val452fs (1) |
|  |  | Uncertain significance (n) | None |
|  |  | Not provided (n) | p.Arg220fs (1), p.Asn1473fs (1), p.Asn1531fs (1), p.Asn1782fs (1), p.Asn741fs (1), p.Asp1486fs (1), p.Gln1035Ter (1), p.Gln1303Ter (1), p.Gln222fs (1), p.Gln264Ter (1), p.Glu1374Ter (2), p.Glu1577fs (1), p.Glu1577Ter (1), p.Glu211Ter (1), p.Glu225Ter (1), p.Glu460Ter (1), p.Glu918Ter (1), p.Glu941Ter (1), p.Gly1094fs (1), p.Gly1288Ter (1), p.Gly797fs (1), p.Leu1489fs (1), p.Leu1669Ter (1), p.Leu456fs (1), p.Lys1085Ter (1), p.Lys1308Ter (1), p.Lys1370Ter (2), p.Lys1518Ter (1), p.Lys2689fs (1), p.Lys993Ter (1), p.Ser1861Tyr (1), p.Ser671fs (1), p.Ser844fs (1), p.Tyr1147Ter (1), p.Tyr935fs (1), p.Val1472fs (1) |
| PIK3CA | 32 (33) | Pathogenic/ Likely pathogenic (n) | p.Arg108His (1), p.Arg88Gln (3), p.Asn345Lys (1), p.Gln546His (1), p.Glu542Lys (3), p.Glu545Gly (1), p.Glu545Lys (11), p.His1047Arg (6), p.His1047Leu (2), p.Met1043Ile (1) |
|  |  | Uncertain significance (n) | p.Asn1044Lys (1) |
|  |  | Not provided (n) | p.Arg357Gln (1), p.Arg93Trp (1), |
| FBXW7 | 18 (21) | Pathogenic/ Likely pathogenic (n) | p.Arg465Cys (2), p.Arg465His (3), p.Arg479Gln (3), p.Arg505Cys (2), p.Arg505Leu (1) |
|  |  | Uncertain significance (n) | None |
|  |  | Not provided (n) | p.Arg224Ter (1), p. p.Arg689Trp (1), p.Asn598fs (2), p.Ile605fs (1), p.Pro201fs (1), Ser582Leu (3), p.Ser625fs (1) |
| SMAD4 | 17 (19) | Pathogenic/ Likely pathogenic (n) | p.Arg361Cys (2), p.Arg361His (6), p.Arg361Ser (1), p.Glu330Ala (1), p.His530fs (1) |
|  |  | Uncertain significance (n) | p.Gln549Ter (1) |
|  |  | Not provided (n) | p.Asp351Gly (1), p.Asp396fs (1), p.Glu520Ter (1), p.Gly395fs (1), p.Gly419Arg (1), p.Pro356Leu (2) |
| ATM | 14 (38) | Pathogenic/ Likely pathogenic (n) | p.Arg1875Ter (1), p.Arg250Ter (1), p.Arg2993Ter (1), p.Arg3008His (1), p.Gly301fs (1), p.Lys2811fs (2), p.Pro1566fs (1), p.Ser1281fs (1), p.Ser1799fs (1) |
|  |  | Uncertain significance (n) | p.Arg23Gln (1), p.Arg337Cys (1), |
|  |  | Not provided (n) | p.Ala1942fs (3), p.Ala2225fs (1), p.Arg1619fs (1), p.Asn1062fs (1), p.Asn2586fs (1), p.Gln268fs (1), p.Gln446Ter (1), p.Glu376Ter (1), p.Gly2083Ter (1), p.Ile1659Ter (1), p.Ile1960fs (1), p.Ile598fs (1), p.Lys902fs (1), p.Phe2908fs (1), p.Phe2927fs (2), p.Phe492fs (1), p.Phe771fs (1), p.Pro453fs (1), p.Ser1360fs (1), p.Ser2581fs (1), p.Thr491fs (2), p.Trp308Ter (1) |
| TET2-AS1 | 13 (18) | Pathogenic/ Likely pathogenic (n) | None |
|  |  | Uncertain significance (n) | None |
|  |  | Not provided (n) | p.Ala893fs (1), p.Arg1404Ter (1), p.Arg544Ter (1), p.His222fs (1), p.Leu1276fs (1), p.Leu1457Ter (1), p.Lys1669fs (1), p.Phe329fs (1), p.Phe868Leu (9), p.Thr624fs (1) |
| PTEN | 9 (12) | Pathogenic/ Likely pathogenic (n) | p.Arg130Gln (1), p.Arg173His (1), p.Gln261Ter (1), p.Gln298Ter (1), p.Glu7Ter (1), p.Ile101fs (1) |
|  |  | Uncertain significance (n) | p.Pro38Thr (1) |
|  |  | Not provided (n) | p.Ala151Thr (1), p.Asn340fs (1), p.Lys144Thr (1), p.Tyr177His (1), p.Tyr68Phe (1) |
| BRCA2 | 7 (30)^b^ | Pathogenic/ Likely pathogenic (n) | p.Glu2183Ter (1), p.Glu510Ter (1), p.Ile2278fs (2), p.Lys1132fs (1), p.Val726fs (2) |
|  |  | Uncertain significance (n) | p.Arg3384Ter (1) |
|  |  | Benign (n) | p.Asn289His (1), p.Asn991Asp (1), p.Val2466Ala (1) |
|  |  | Not provided (n) | p.Asn2463fs (1), p.Gln2354fs (1), p.Gln2655fs (3), p.Lys697fs (1), p.Pro1702fs (1), p.Pro2246fs (1), p.Val1532fs (1), |

^a^Of 75 patients, 5 showed amplification.

^b^Of 30 cases, 6 had synonymous mutations, and 4 had intronic mutations.

Each SNV was classified according to the ClinVar database (<https://www.ncbi.nlm.nih.gov/clinvar/>).

**Table A2. Mutation spectrum of driver genes in the TCGA cohort**

| **Gene** | **Case (Variants)** | **Variation** |
| --- | --- | --- |
| APC | 160 (251^a^) | p.1219_1219del (1), p.1275_1275del (1), p.1289_1291del (1), p.1291_1291del (1), p.1293_1293del (1), p.A1089V (1), p.A2028T (1), p.A209V (1), p.A2D (1), p.A513V (1), p.C1252X (1), p.C1392S (1), p.C1392X (1), p.C507X (1), p.D2501G (1), p.D756G (2), p.E1191X (2), p.E1239X (1), p.E1268X (1), p.E1277fs (1), p.E1277X (1), p.E1288X (3), p.E1291X (2), p.E1304X (1), p.E1335X (1), p.E1356X (3), p.E1361X (1), p.E1379X (1), p.E1390X (2), p.E146K (2), p.E1518X (2), p.E1555fs (1), p.E1560fs (1), p.E190fs (1), p.E200X (1), p.E829fs (2), p.E923X (1), p.E966X (1), p.F1336fs (2), p.F2766C (1), p.G1270X (1), p.G1312fs (1), p.G549X (1), p.H1472fs (1), p.H289fs (1), p.I1236N (1), p.I1286fs (1), p.I1644I (1), p.I2497V (1), p.K1067X (1), p.K1352X (1), p.K2174T (1), p.K652X (2), p.K921X (1), p.K975X (1), p.L1471X (1), p.L235X (1), p.L478fs (1), p.M1395fs (1), p.N1143K (1), p.P1301fs (1), p.P1351P (1), p.P1453fs (1), p.P1742R (1), p.P2338H (1), p.P2743L (2), p.Q1017X (1), p.Q1023E (1), p.Q1134X (1), p.Q1173X (1), p.Q1210X (1), p.Q1226X (1), p.Q1242X (1), p.Q1244fs (1), p.Q1276X (3), p.Q1285X (1), p.Q1310X (1), p.Q1320X (2), p.Q1349X (4), p.Q1360fs (1), p.Q1360X (3), p.Q1378X (1), p.Q1388X (4), p.Q1411X (1), p.Q1451X (1), p.Q246X (1), p.Q394X (1), p.Q462X (1), p.Q524X (1), p.Q636X (1), p.Q649X (1), p.Q739X (1), p.Q883X (1), p.Q960X (2), p.R1096X (6), p.R1381H (1), p.R1417fs (1), p.R1432X (19), p.R1450X (1), p.R216X (7), p.R223X (5), p.R2311I (1), p.R232X (4), p.R2413K (1), p.R265X (2), p.R284X (3), p.R330X (1), p.R387X (1), p.R481X (2), p.R499X (1), p.R536X (2), p.R546X (6), p.R554X (1), p.R640W (1), p.R787X (6), p.R827H (1), p.R858X (11), p.R906X (1), p.S1145fs (1), p.S1180X (1), p.S1263X (1), p.S1264X (1), p.S1297X (2), p.S1328X (4), p.S1380fs (1), p.S1382L (2), p.S1382X (1), p.S1393fs (1), p.S1401N (1), p.S1403fs (2), p.S2011Y (1), p.S2411F (1), p.S2464Y (1), p.S569S (1), p.S578X (1), p.S695X (1), p.S752X (1), p.S836fs (1), p.S945fs (1), p.S960fs (1), p.T1283fs (1), p.T1380fs (1), p.T1420fs (1), p.T1469fs (1), p.T646T (1), p.T916fs (1), p.V1334fs (1), p.V291fs (1), p.V539A (1), p.V579fs (1), p.V812fs (1), p.Y1165X (1), p.Y1358X (1), p.Y917X (2), p.Y935_N936delinsX (1), p.Y938C (1) |
| TP53 | 122 (126) | p.122_122del (1), p.173_173del (1), p.177_183del (1), p.212_212del (1), p.289_291del (1), p.98_99del (1), p.A138V (1), p.A159V (1), p.C124G (1), p.C135S (1), p.C141R (1), p.C141S (1), p.C141Y (1), p.C176F (1), p.C176X (1), p.C238Y (1), p.C275Y (1), p.D207N (1), p.E154K (1), p.E258X (1), p.E285X (1), p.E286G (1), p.F109C (1), p.F113C (1), p.G154C (1), p.G244D (2), p.G245D (1), p.G245S (4), p.G266X (1), p.H178fs (1), p.I195T (1), p.I251F (1), p.I63fs (1), p.K132N (1), p.K132T (1), p.K187fs (1), p.L130F (1), p.L194H (1), p.L35fs (1), p.M237I (1), p.P151H (1), p.P152R (1), p.P278A (1), p.P278R (1), p.P27fs (1), p.P322fs (1), p.P60fs (1), p.Q199fs (1), p.Q331H (1), p.Q331X (1), p.R175C (2), p.R175H (15), p.R196X (3), p.R213X (7), p.R248Q (1), p.R248W (9), p.R267W (1), p.R273C (4), p.R273H (7), p.R282W (3), p.R306X (4), p.R335fs (2), p.R337C (1), p.R342X (1), p.S127P (1), p.S33fs (1), p.T125M (2), p.T125T (1), p.T231T (1), p.V143A (1), p.V173M (1), p.V274L (2), p.Y205C (1), p.Y236X (1) |
| KRAS | 94 (95^b^) | p.A146T (8), p.A146V (1), p.E98X (1), p.G12A (1), p.G12C (6), p.G12D (31), p.G12R (1), p.G12S (4), p.G12V (23), p.G13D (10), p.K117N (2), p.Q22K (1), p.Q61L (2), p.R68S (1) |
| FBXW7 | 39 (50) | p.321_322del (1), p.A235A (1), p.D440N (1), p.D440Y (1), p.D520Y (2), p.E112A (1), p.E113D (1), p.E207V (1), p.G357R (1), p.H415P (1), p.K159Q (2), p.K167T (2), p.P4P (1), p.Q501X (1), p.R13X (1), p.R14Q (2), p.R259X (1), p.R287X (2), p.R385C (4), p.R385H (8), p.R393fs (1), p.R399X (1), p.R425C (3), p.R425G (1), p.R425H (1), p.R578Q (1), p.R578X (2), p.R609W (1), p.R99H (1), p.S396R (1), p.S502L (2), p.T144T (1) |
| PIK3CA | 34 (42) | p.D258N (1), p.D350G (1), p.D925D (1), p.E542K (2), p.E545A (1), p.E545G (1), p.E545K (8), p.E545Q (1), p.E81K (1), p.G106R (1), p.G364R (1), p.H1047R (5), p.H1047R (4), p.M1043I (1), p.N345K (2), p.P471L (1), p.Q546K (1), p.R357Q (1), p.R777M (1), p.R88Q (4), p.R951C (1), p.T1052K (1), p.V344A (1), p.V344G (1), p.V955V (1), p.Y1021C (1) |
| SMAD4 | 26 (31) | p.30_30del (1), p.36_45del (1), p.A327V (1), p.C324R (1), p.D355G (1), p.D537G (1), p.D537V (1), p.D537Y (1), p.E134K (1), p.E33X (1), p.E520X (1), p.G510R (1), p.G65E (1), p.L104F (1), p.L533R (1), p.L57V (1), p.P356L (1), p.R361H (8), p.R496H (1), p.R97H (1), p.S357P (1), p.V136A (1), p.V437D (1), p.Y513X (1) |
| ATM | 25 (50) | p.2693_2694del (1), p.A1024T (1), p.A920V (1), p.C1168C (1), p.D2721N (1), p.E1666X (1), p.E1822X (1), p.E2039K (1), p.E2676X (1), p.F1837fs (1), p.F2140V (1), p.F2839L (1), p.G2425fs (1), p.G2891R (1), p.H2038H (1), p.H2872R (1), p.K477N (1), p.L1217M (2), p.L1408I (1), p.L1488L (2), p.L2077I (1), p.L2147I (2), p.L2251I (2), p.L2557V (1), p.L822V (1), p.L942I (2), p.N2435K (1), p.P2353T (1), p.Q1117P (1), p.Q499E (1), p.R1150I (2), p.R1466X (2), p.R1489H (1), p.R1730X (3), p.R2060H (1), p.R250X (2), p.R2598Q (1), p.S614N (1), p.T2771I (1), p.W2769X (1) |
| BRAF | 21 (22) | p.F247L (1), p.K205Q (1), p.V600E (20) |
| NRAS | 20 (21) | p.E132K (1), p.G12A (1), p.G12C (3), p.G12D (2), p.G13R (2), p.Q61H (1), p.Q61K (7), p.Q61L (2), p.Q61R (1), p.R164C (1) |

*^a^One patient has no data on amino acid changes.*

*^b^Three patients have no data on amino acid changes.*

Each SNV was classified according to the ClinVar database (<https://www.ncbi.nlm.nih.gov/clinvar/>).

**Table A3. Mutation spectrum of driver genes in the MSKCC cohort**

| **Gene** | **Case (variants)** | **Variation (n ≥ 5)** |
| --- | --- | --- |
| APC | 737 (1203) | p.R876* (44), p.R1450* (41), p.R216* (33), p.T1556Nfs*3 (31), p.R213* (30), p.E1309Dfs*4 (29), p.R232* (24), p.R283* (21), p.S1465Wfs*3 (21), p.Q1367* (16), p.Q1429* (16), p.R1114* (15), p.R554* (15), p.R499* (14), p.R805* (14), p.Y935* (14), p.E1322* (12), p.E1353* (11), p.Q1406* (11), p.R564* (11), p.E1309* (10), p.E1397* (10), p.Q1338* (10), p.R302* (10), p.E1306* (9), p.E1379* (9), p.P1319Lfs*2 (8), p.Q1378* (8), p.E1345* (7), p.L1488Yfs*19 (7), p.Q1303* (7), p.I1307Nfs*8 (6), p.K534* (6), p.S1315* (6), p.S1465Rfs*9 (6), p.E1408* (5), p.E1464Vfs*8 (5), p.E941* (5), p.S1346* (5) |
| TP53 | 710 (780) | p.R175H (67), p.R273H (40), p.R248Q (35), p.R282W (34), p.R273C (32), p.G245S (28), p.R213* (28), p.R248W (22), p.R342* (18), p.R196* (16), p.R306* (9), p.P152L (8), p.G266E (7), p.M237I (7), p.V173L (7), p.X307_splice (7), p.P151S (6), p.R158H (6), p.R209Kfs*6 (6), p.X331_splice (6), p.C176F (5), p.R337C (5), p.S127F (5), p.S241F (5), p.T125M (5), p.X126_splice (5) |
| KRAS | 432 (447) | p.G12D (122), p.G12V (87), p.G13D (77), p.G12C (31), p.A146T (29), p.G12A (22), p.G12S (19), p.Q61H (9), p.G12R (7), p.K117N (7), p.A146V (6), p.Q61K (5) |
| PIK3CA | 190 (219) | p.E545K (52), p.E542K (29), p.H1047R (28), p.Q546K (9) |
| SMAD4 | 153 (170) | p.R361H (19), p.R361C (13), p.D537G (7), p.A118V (5), p.E520* (5) |
| FBXW7 | 114 (130) | p.R465C (13), p.R465H (13), p.R505C (13), p.S668Vfs*39 (6), p.R278* (5) |
| BRAF | 107 (128) | p.V600E (76), p.D594G (6) |
| ATM | 73 (101) | p.R337C (7), p.R3008C (5) |
| NRAS | 44 (44) | p.Q61K (9), p.G12D (7) |

Each SNV was classified according to the ClinVar database (<https://www.ncbi.nlm.nih.gov/clinvar/>)

**Table A4. Correlation of common gene mutations with tumor stages in TCGA cohort**

| **Mutations** | **T stage^a^** | | **N stage^b^** | | **M stage^c^** | | **Stage^d^** | |
| --- | --- | --- | --- | --- | --- | --- | --- | --- |
|  | **1, 2** | **3, 4** | **0** | **1, 2** | **0** | **1** | **1, 2** | **3, 4** |
| **KRAS** | p = 0.7224 | | p = 0.8708 | | p = 0.6389 | | p = 0.5531 | |
| Negative | 55 | 202 | 143 | 113 | 184 | 31 | 134 | 119 |
| Positive | 39 | 160 | 113 | 85 | 145 | 29 | 108 | 84 |
| **NRAS** | p = 0.3883 | | p = 0.8406 | | p = 0.7946 | | p = 1 | |
| Negative | 85 | 339 | 239 | 183 | 304 | 55 | 225 | 188 |
| Positive | 9 | 23 | 17 | 15 | 25 | 5 | 17 | 15 |
| **BRAF** | p = 0.6445 | | **p = 0.00398** | | p = 0.05844 | | **p = 0.00195** | |
| Negative | 80 | 317 | 212 | 183 | 281 | 57 | 199 | 188 |
| Positive | 14 | 45 | 44 | 15 | 48 | 3 | 43 | 15 |
| **PIK3CA** | p = 0.6271 | | **p = 0.0217** | | p = 0.1388 | | **p = 0.00253** | |
| Negative | 70 | 258 | 173 | 154 | 229 | 48 | 161 | 162 |
| Positive | 24 | 104 | 83 | 44 | 100 | 12 | 81 | 41 |
| **TP53** | p = 0.06349 | | **p = 0.00025** | | p = 0.0747 | | **p = 0.00016** | |
| Negative | 42 | 122 | 111 | 52 | 125 | 15 | 106 | 53 |
| Positive | 52 | 240 | 145 | 146 | 204 | 45 | 136 | 150 |
| **APC** | p = 0.6315 | | p = 0.3294 | | p = 0.4598 | | p = 0.2612 | |
| Negative | 18 | 80 | 60 | 38 | 72 | 10 | 57 | 38 |
| Positive | 76 | 282 | 196 | 160 | 257 | 50 | 185 | 165 |
| **SMAD4** | p = 0.3371 | | p = 0.09645 | | p = 1 | | p = 0.243 | |
| Negative | 84 | 307 | 226 | 163 | 282 | 51 | 212 | 169 |
| Positive | 10 | 55 | 30 | 35 | 47 | 9 | 30 | 34 |
| **FBXW7** | p = 0.2788 | | p = 0.25 | | p = 0.07132 | | p = 0.2284 | |
| Negative | 81 | 292 | 204 | 167 | 260 | 54 | 192 | 171 |
| Positive | 13 | 70 | 52 | 31 | 69 | 6 | 50 | 32 |
| **ATM** | p = 0.8081 | | p = 0.2109 | | **p = 0.00766** | | p = 0.2312 | |
| Negative | 80 | 314 | 216 | 176 | 276 | 58 | 204 | 180 |
| Positive | 14 | 48 | 40 | 22 | 53 | 2 | 38 | 23 |

*^a^Three patients have no available data.*

*^b^Five patients have no available data.*

*^c^A total of 70 patients have no available data.*

*^d^A total of 14 patients have no available data.*

**Table A5. Classification of genes according to median VAF**

| **Low VAF genes** | **High VAF genes** |
| --- | --- |
| BRCA1, CDKN2A, BAP1, TSC2, TSC1, POLE, PALB2, ATM, SMARCA4, PIK3R1, NOTCH1, NF1, NF2, STK11, MSH2, LOC100507346,PTCH1 | PTCH1, VHL, CDH1, PTEN, RB1, FBXW7, BRCA2, PIK3CA, AKT1, ARID1A, BRAF, APC, KRAS, TET2, TET2-AS1, SMAD4, TP53, ERBB2, ATRX |

**Table A6. Correlation of MSI status according to the clinicopathological data and KRAS, NRAS, PIK3CA, and BRAF mutations in the TCGA cohort**

| **Clinicopathological parameters** | **MSI status^a^** | |
| --- | --- | --- |
|  | **MSS/MSI-L** | **MSI-H** |
| **Age^b^** | p = 0.1627 | |
| ≥60 years | 262 | 50 |
| <60 years | 127 | 15 |
| **Sex** | p = 0.3295 | |
| Male | 204 | 29 |
| Female | 188 | 36 |
| **T stage^c^** | p = 1 | |
| 1, 2 | 81 | 13 |
| 3, 4 | 308 | 52 |
| **N stage^d^** | **p = 5.222e-05** | |
| 0 | 202 | 52 |
| 1, 2 | 185 | 13 |
| **M stage^e^** | p = 0.05575 | |
| 0 | 279 | 50 |
| 1 | 57 | 3 |
| **Stage^f^** | **p = 7.857e-06** | |
| 1, 2 | 189 | 51 |
| 3, 4 | 191 | 12 |
| **Tumor site^g^** | **p = 4.554e-14** | |
| Right colon | 125 | 51 |
| Left colon | 239 | 7 |
| **KRAS mutation** | **p = 0.03194** | |
| Absent | 212 | 45 |
| Present | 180 | 20 |
| **NRAS mutation** | p = 0.6002 | |
| Absent | 363 | 62 |
| Present | 29 | 3 |
| **PIK3CA mutation** | **p = 0.03334** | |
| Absent | 289 | 39 |
| Present | 103 | 26 |
| **BRAF mutation** | **p < 2.2e-16** | |
| Absent | 371 | 27 |
| Present | 21 | 38 |

*^a^Two cases have no available data.*

*^b^Three cases have no available data.*

*^c^Three cases have no available data.*

*^d^Five cases have no available data.*

*^e^A total of 70 cases have no available data.*

*^f^A total of 14 cases have no available data.*

*^g^Three cases have no available data.*

**Table A7. The MMR protein and BRAF and MMR gene mutation status in MSI-H group**

| **Case no.** | **Age** | **Sex** | **Past history**  **(related cancer)** | **Family history**  **(related cancer)** | **MLH1 IHC^a^** | **MLH2 IHC** | **PMS2 IHC** | **MSH6 IHC** |
| --- | --- | --- | --- | --- | --- | --- | --- | --- |
| 1 | 59 | M | N |  | Positive | Positive | Positive | Negative |
| 2 | 84 | M |  |  | Negative | Positive | Negative | Positive |
| 3 | 54 | F | Breast cancer |  | Positive | Negative | Positive | Negative |
| 4 | 26 | M |  |  | Negative | Positive | Negative | Positive |
| 5 | 54 | F |  | CRC (sibling) | Negative | Positive | Negative | Positive |
| 6 | 57 | M |  | CRC (father, older brothers) | Negative | Positive | NA | NA |
| 7 | 67 | F |  | Stomach cancer (son) | Positive | Focally positive | Positive | Focally positive |
| 8 | 46 | M | Stomach cancer | Panceratic cancer (father) | Negative | Positive | Negative | Positive |
| 9 | 69 | F | Breast cancer |  | Negative | Positive | Negative | Positive |
| 10 | 30 | M |  |  | Negative | Positive | Negative | Positive |

^a^IHC; immunohistochemistry

**Table A7** *(continued)*

| **Case no.** | **BRAF**  **status** | **MLH1 status** | **MLH2 status** | **PMS2 status** | **MSH6 status** |
| --- | --- | --- | --- | --- | --- |
| 1 | Mutant | Wild | Wild | Wild | Mutant |
| 2 | Wild | Wild | Mutant | Wild | Wild |
| 3 | Wild | Wild | Mutant | Wild | Mutant |
| 4 | Wild | Wild | Wild | Wild | Wild |
| 5 | Wild | Wild | Wild | Wild | Wild |
| 6 | Wild | Mutant | Wild | Wild | Wild |
| 7 | Wild | Wild | Wild | Wild | Wild |
| 8 | Wild | Wild | Wild | Wild | Wild |
| 9 | Wild | Wild | Wild | Wild | Wild |
| 10 | Wild | Wild | Wild | Wild | Wild |
